# Alteration of the gut microbiota profile in children with autism spectrum disorder in China

**DOI:** 10.3389/fmicb.2023.1326870

**Published:** 2024-02-13

**Authors:** Hui Li, Wei Guo, Sijie Li, Bishao Sun, Ningshan Li, Dongjing Xie, Zongming Dong, Dan Luo, Wei Chen, Weihua Fu, Ji Zheng, Jingzhen Zhu

**Affiliations:** ^1^Department of Ultrasound, Xinqiao Hospital, Army Medical University, Chongqing, China; ^2^Stroke Center, Puyang People's Hospital, Puyang, China; ^3^Department of Pediatrics, Xinqiao Hospital, Army Medical University, Chongqing, China; ^4^Department of Urology, Urologic Surgery Center, Xinqiao Hospital, Army Medical University, Chongqing, China; ^5^Department of Neurology, Xinqiao Hospital, Army Medical University, Chongqing, China; ^6^Department of Neurology, Yunyang People's Hospital, Yunyang, China

**Keywords:** gut microbiota, autism spectrum disorder, Chinese children, metabolic pathways, gut-brain axis

## Abstract

**Background:**

Autism spectrum disorder (ASD) is associated with alterations in the gut microbiome. However, there are few studies on gut microbiota of children with ASD in China, and there is a lack of consensus on the changes of bacterial species.

**Purpose:**

Autism spectrum disorder (ASD) is associated with alterations in the gut microbiome. However, there are few studies on gut microbiota of children with ASD in China, and there is a lack of consensus on the changes of bacterial species.

**Methods:**

We used 16S rRNA sequencing to analyze ASD children (2 to 12 years), HC (2 to 12 years).

**Results:**

Our findings showed that the α-diversity, composition, and relative abundance of gut microbiota in the ASD group were significantly different from those in the HC groups. Compared with the HC group, the α-diversity in the ASD group was significantly decreased. At the genus level, the relative abundance of g_Faecalibacterium, g_Blautia, g_Eubacterium_eligens_group, g_Parasutterella, g_Lachnospiraceae_NK4A136_group and g_Veillonella in ASD group was significantly increased than that in HC groups, while the relative abundance of g_Prevotella 9 and g_Agathobacter was significantly decreased than that in HC groups. In addition, KEGG pathway analysis showed that the microbial functional abnormalities in ASD patients were mainly concentrated in metabolic pathways related to fatty acid, amino acid metabolism and aromatic compound metabolism, and were partially involved in neurotransmitter metabolism.

**Conclusion:**

This study revealed the characteristics of gut microbiota of Chinese children with ASD and provided further evidence of gut microbial dysbiosis in ASD.

## Introduction

Autism spectrum disorder (ASD) refers to a group of complex neurodevelopmental disorders that typically present in early childhood and are characterized by stereotyped behavior, language, and social interaction disorders (Valentino et al., 2021). The prevalence of ASD has increased significantly in recent decades, affecting 1 in 59 children in the United States (Maenner et al., 2020) and approximately 39.23/10000 Chinese children aged 1.6–8 years (Wang et al., 2018). Patients with ASD require lifelong treatment and intervention, making it a serious public health concern (Joon et al., 2021). Both genetic and environmental factors are believed to be potential triggers for ASD (Wang et al., 2016; Modabbernia et al., 2017; Lord et al., 2018), with abnormal gut microbiota being an important environmental factor. Gastrointestinal problems are common in children with ASD, affecting 9–91% of cases (Ferguson et al., 2019) and often correlating with the severity of ASD (Adams et al., 2011).

Research has shown that the human gut microbiota plays a crucial role in influencing the brain through a communication pathway, called the gut-brain axis (Fowlie et al., 2018; Iannone et al., 2019; Lacorte et al., 2019; Sharon et al., 2019; Srikantha and Mohajeri, 2019). Changes in the gut microbiota can regulate gastrointestinal physiology, immune function, and behavior through the gut-microbiota-brain axis (Li et al., 2019; Sharon et al., 2019; Tomova et al., 2020; Nogay and Nahikian-Nelms, 2021). Germ-free animal studies have demonstrated that the gut microbiota shapes the brain by influencing synaptic signaling and gene transmission (Sanlier and Kocabas, 2021). *Lactobacillus reuteri* treatment has been shown to selectively reverse social deficits in mouse models of ASD, further highlighting the involvement of gut microbiota in the pathogenesis of ASD (Sgritta et al., 2019). While several human studies have shown alterations in the gut microflora in ASD, there is little consensus on the specific bacterial species involved. Conflicting results have been reported in different population studies, with some showing increased bacterial diversity (Wan et al., 2022), others showing no change (Kandeel et al., 2020), and still others showing a decrease (Dan et al., 2020) compared with healthy controls. Some studies have found that potentially harmful microbiota, such as Clostridium (Wang et al., 2019), Sutterellaceae (de Angelis et al., 2013), and Enterobacteriaceae (Ding et al., 2020), were more abundant in children with ASD, while Firmicutes (Liu et al., 2019) and Prevotellaceae (Pulikkan et al., 2018) were decreased in the ASD group. However, other studies have shown the opposite results (Rose et al., 2018; Zou et al., 2020; Ye et al., 2021). Studies have also found that Clostridium and Bacteroidetes/Firmicutes ratios were increased in ASD children with functional gastrointestinal disease (Luna et al., 2017), while a study in Slovakia showed increased numbers of Lactobacillus and a significant decrease in the Bacteroidetes/Firmicutes ratio (Tomova et al., 2015). Another study suggested that decreased Bifdobacteria abundance might lead to reduced folate production in individuals with ASD, so abnormal folate metabolism might be associated with ASD (Frye et al., 2017). In conclusion, while the results on gut microbiota and ASD are still inconsistent and mainly focused on Western populations, there is evidence to suggest that altered gut microbiota is associated with ASD. Further research is needed to better understand the role of gut microbiota in ASD and to identify non-invasive biomarkers for early diagnosis.

Given the inconsistent findings on the gut microbiota in ASD and the limited research on Chinese populations, it is important to investigate the changes in gut microbiota in Chinese children with ASD. Based on fecal 16S ribosomal DNA (rDNA) sequencing data from 957 children with ASD and 161 healthy controls in China, we aimed to determine the taxonomic composition of gut microbiota of children with ASD and the changes in the gut microbiota compared with healthy controls. Additionally, we identified potential biomarkers of bacterial species that could serve as non-invasive tools for early diagnosis of ASD.

## Materials and methods

### Ethics statement

This study was approved by the Ethics Committee of Second Affiliated Hospital, Army Medical University (Ethics NO. 2023–001-01). Written informed consents were signed by all the children’s legal guardians prior to the study. The clinical trial number: ChiCTR2300074832.

### Study subject recruitment and fecal sample collection

In total, 1,118 participants from 12 provinces of China including 957 participants with clinical definition as ASD (aged between 2 and 12 years, average age 4.6) and 161 health children (HC) (aged between 2 and 12 years, average age 4.8) were recruited (Supplementary Table S1).

All children with ASD participating in this study were enrolled from social groups of unrelated families with autism (Participants had been diagnosed with ASD according to DSM-5 (Diagnostic and Statistical Manual of Mental Disorders - 5th Edition) before enrollment). The exclusion criteria included diseases such as depressive disorder, cerebral palsy, schizophrenia, bipolar disorder, significant sensory impairment, and clinically significant inflammatory conditions.

The control group of children were recruited from kindergartens and primary school. Children with psychiatric conditions (such as depressive disorder, schizophrenia, and bipolar disorder) were excluded on the basis of entrance examinations and parent interviews.

All subjects had not taken antibiotics, antipsychotics, probiotics, and prebiotics in the month prior to the sample collection. Feces samples were used to analyze the genes associated with microbiota. Supplementary Figure S1 presents the study flowchart.

Feces were collected by parents in hospitals or at home. The feces were collected according to the instructions and delivered immediately at a low temperature. The frozen feces were transported overnight with dry ice to CNNC Yili (Tianjin) Medical Laboratory Co., Ltd., where they were frozen at −80°C until DNA was extracted.

### 16S rRNA sequencing and analysis

DNA from intestinal contents was prepared from subjects in the ASD and HC groups using a fecal DNA extraction kit (DP712, Tiangen Company, Beijing, China). The analysis was repeated with three biological replicates in each group. DNA quality was monitored on 1% agarose gels. The hypervariable V3-V4 region of the 16S-rDNA gene was amplified by PCR using the primers 338F (5’-ACTCCTACGGGAGGCAGCAG-3′) and 806R (5’-GGACTACHVGGGTWTCTAAT-3′), where the barcode was an eight-base sequence unique to each sample. PCR was performed in 30-μL reactions composed of 15 μL of Phusion^®^ High-Fidelity PCR Master Mix (New England Biolabs), 0.2 μM forward and reverse primers, and 10 ng of template DNA. The thermal cycle consisted of an initial denaturation step at 98°C for 1 min, followed by 30 cycles of denaturation at 98°C for 10 s, annealing at 50°C for 30 s, and elongation at 72°C for 30 s. Finally, the reactions were incubated at 72°C for 5 min. PCR products were detected on 2% agarose gels by electrophoresis and then purified using the GeneJET Gel Extraction Kit (Thermo Scientific). Sequencing libraries were generated using the Illumina TruSeq DNA PCR-Free Library Preparation Kit (Illumina, United States) according to the manufacturer’s recommendations, and index codes were added. Sequencing was performed using the Illumina NovaSeq MiSeq PE300 platform (Genecloud Co. Ltd., Chongqing, China). The sequence analysis was performed using the QIIME software package 2 (version 2020.2). The raw data of 16S rRNA sequencing underwent decomposition and quality filtering using version 0.20.0 of fastp, and merged using FLASH version 1.2.7 with the following merging conditions: (i) Any reads with an average quality score < 20 in a 50 bp sliding window were truncated at any position. Reads shorter than 50 bp were discarded, as well as reads containing ambiguous characters. (ii) Assembly was performed only based on overlapping sequence lengths greater than 10 bp, with a maximum mismatch ratio of 0.2 in the overlapping region. Reads that could not be assembled were discarded. (iii) Samples were differentiated based on barcodes and primers, and the sequence orientation was adjusted. Barcodes were matched exactly, while primer matching allowed for 2 nucleotide mismatches. Using the MOTHUR workflow, cluster effective tags with a similarity of ≥97% into operational taxonomic units (OTUs). The representative sequence within each cluster was selected as the tag sequence with the highest abundance. Bacterial alpha diversity was determined based on OTU analysis of samples and expressed as Chao index, Shannon index and Simpson index, which were calculated using the R program package “vegan.” Principal coordinate analysis (pCoA) was performed using the R package^1^ to represent the β diversity of the microbiome. Using the phyloseq package to calculate Bray-Curtis metric distances, UniFrac distances, and weighted UniFrac distances. The wilcoxon rank sum test was used to compare the differences of bacterial phyla, classes, orders, families and genera between the two groups. The linear discriminant analysis (LDA) effect size (LEfSe) method was used to analyze the bacterial community dominance between groups.^2^ LEfSe and Kruskal−Wallis rank sum test (*p* < 0.05) to identify features with significant differences in abundance levels between specified taxa, and to evaluate the effect size of each feature using LDA (LDA score (log10) =3.5 as the cutoff value). The R language mixOmics package was used to calculate Pearson correlation coefficient, and the correlation coefficient *r*^2^ and *p* value of differential gut microbiota and differential metabolites were calculated. The metabolic pathways of intestinal microorganisms were analyzed based on the Kyoto Encyclopedia of Genes and Genomes (KEGG) database.^3^ We evaluated the metabolic pathway enrichment of the two groups. The metabolic pathway was considered enriched, while when the p value of the metabolic pathway was <0.05. Correlation analysis was performed by calculating Pearson’s correlation coefficient using the R language mixOmics package to calculate the correlation coefficient *r*^2^ and the *p* value of differentially bacterial and differentially metabolites.

The raw data of 16S rRNA sequence of this paper have been deposited in the Genome sequence Archive (Genomics, Proteomics & Bioinformatics 2021) of the National Genomics Data Center (Nucleic Acids Res 2022) and the National Center for Biological Information of China/Beijing Institute of Genomics, Chinese Academy of Sciences (GSA - human: HRA004410), and can be in open access at https://ngdc.cncb.ac.cn/gsa-human.

### Co-occurrence network analysis

To understand the correlations among different genera, we constructed co-occurrence network based on the 16S rRNA data (Frye et al., 2017; Wang et al., 2018). The bacterial correlations in the ASD and HC samples were analyzed, respectively, according to the relative abundance of each genus using Spearman’s correlation coefficient to construct the co-occurrence network. The significant correlated genus (*p* < 0.05, *r* ≥ 0.7) were visualized by Gephi 0.9.7 (www.netbeans.org). Then, the similarity between the two network structures was measured by node closeness and shared correlations. Closeness of the nodes was analyzed by Gephi to predicate node centralities in each network. The shared correlations between two groups were defined the edges with the same nodes in two co-occurrence networks. Only genera existed in at least 10% sample were included in the network analysis.

### Statistical analysis

The data was presented as mean ± standard error (SEM). SPSS 26.0 software (SPSS, Inc., Chicago, IL, United States) was used to perform independent sample t test for the two groups of data. A two-tailed Wilcoxon rank-sum test was performed for the analysis of gut microbiota sequencing data by R Project. *p* < 0.05 was considered statistically significant.

## Results

### Differences of microbial diversity between ASD and healthy controls

**Figure 1:**
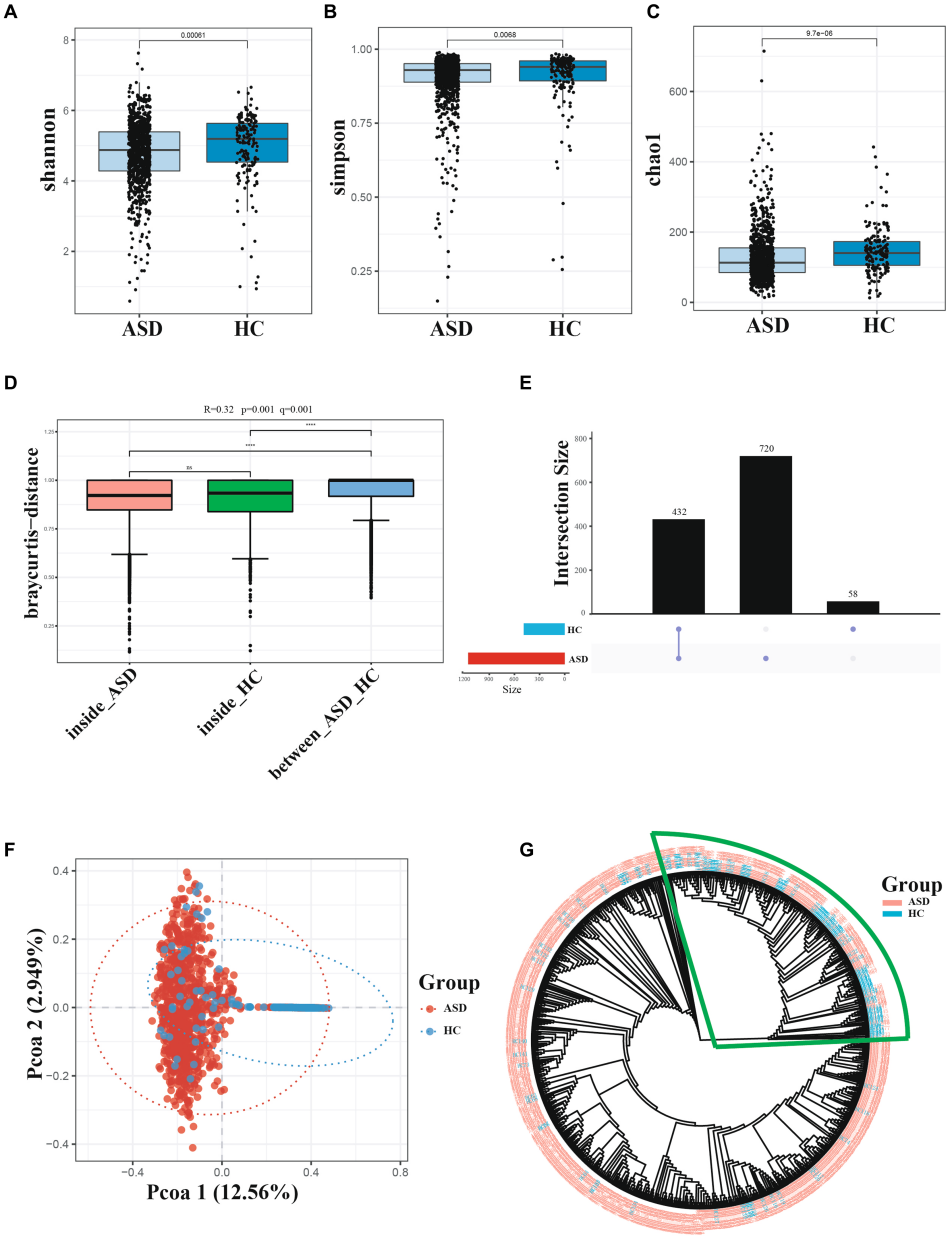
Diversity of gut microbiota are different across ASD and HC. **(A−C)** Index of Alpha diversity (the Shannon, simpson, and Chao1). **(D)** Anosim-analysis results, between represents the difference between groups; others are within groups; the greater the distance is, the greater the difference is; and the thickness is the sample size. **(E)** UpSet plot of intersections and unique among different group. **(F)** pCoA of beta diversity. **(G)** Cluster evolutionary tree analysis. *p < 0.05, **p < 0.01 and ***p < 0.001.

The alpha diversity of gut microbiota was measured using various indices, including the Shannon index, Chao1 index, and Simpson index. The alpha diversity of gut microbiota in the ASD group showed a significant difference when compared with those of the HC groups (Figure 1). Specifically, these diversity indices showed a similar trend, that is, the alpha diversity of gut microbiota was higher in the HC group than in the ASD group (Figures 1A−C, online Supplementary Figure S2). The Anosim-analysis also showed that the differences between HC and ASD groups were significantly greater than those within the group, indicating meaningful grouping (Figure 1D). Figure 1E illustrated that the samples obtained from the ASD group had 1,152 features, while only 490 features were detected in the samples from the HC group, both groups shared a total of 432 features. These findings suggested that a significant number of features were present exclusively in the ASD group, indicating a higher relative abundance of certain species in the gut microbiota of children with ASD compared to healthy children. The pCoA principal component analysis based on bray-curtis clearly showed the microbiota differences between the ASD and HC groups. There were significant differences along the PCO1 axis, accounting for 12.56% of the total variation (Figure 1F). Sample cluster evolutionary tree analysis also effectively clustered the ASD and HC group (Figure 1G). In conclusion, the species richness and diversity of gut microbiota in children with ASD were significantly different from those in HC group.

### Differences of microbial comparison between ASD and healthy controls

**Figure 2:**
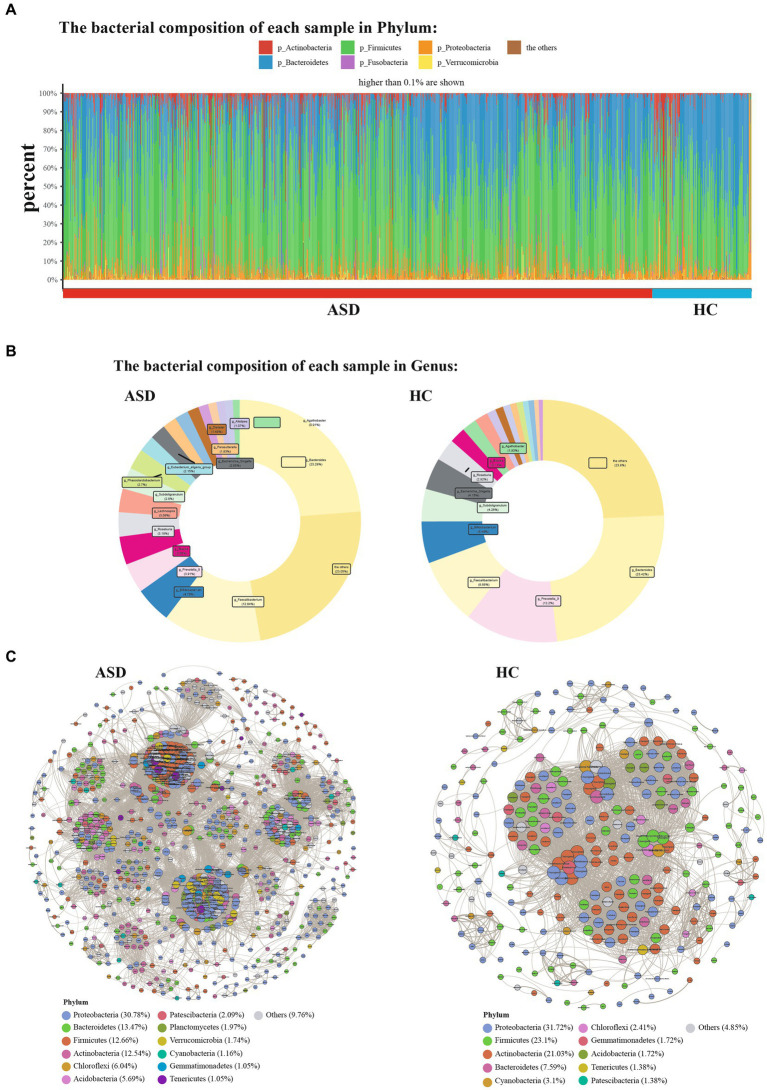
Phylum and Genera of gut microbiota are strikingly different across ASD and HC. **(A)** Relative abundance of the indicated phylum. **(B)** Relative abundance of the indicated genus. **(C)** Genera co-occurrence network between ASD and HC group based on the Spearman correlation algorithms. Each node presents a bacterial genus. The node size indicates the weighted value of each genus per group, and the thickness of the line represents the Spearman coefficient (*p* < 0.05, r ≥ 0.7).

**Figure 3:**
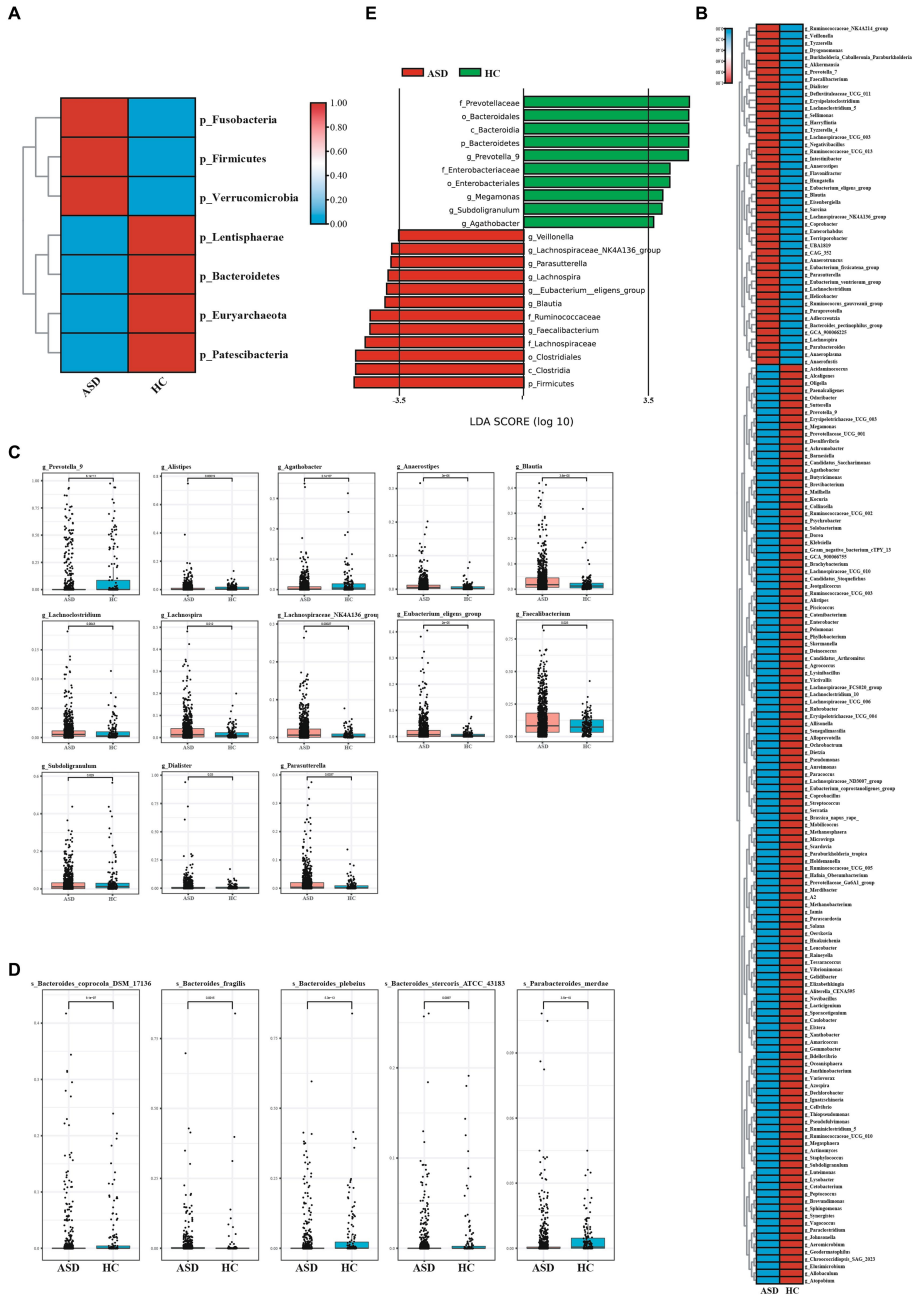
The shift of gut microbiota in ASD and HC. **(A)**Heatmap of selected differentially abundant phylum in ASD and HC (*p* < 0.05). **(B)** Heatmap of selected differentially abundant genus in ASD and HC (*p* < 0.05). **(C,D)** The top 20 of differentially abundant analysis between ASD and HC at the level of genus **(C)** and species **(D)**. **(E)** LDA scores for the bacterial taxa showed different abundant between ASD and HC (LAD score > 3.5) (*p* < 0.05). *p < 0.05, **p < 0.01, and ***p < 0.001.

Next, the composition of gut microbiota in ASD, and HC group was further analyzed. At the phylum level, Firmicutes, Bacteroidetes, Proteobacteria and Actinobacteria were found to be dominant in the different groupings (Figure 2A). In comparison to the HC group, the ASD group exhibited higher levels of Firmicutes and a significantly lower Bacteroidetes/Firmicutes ratio (ASD = 0.54, HC = 0.89). At the genus level, g_Bacteroides, g_Faecalibacterium, g_Prevotella_9, g_Bifidobacterium, g_Phascolarctobacterium and g_Blautia were found to be dominant in the different groupings (Figure 2B). At the phylum level, the ASD group showed a significant decrease in p_Lentisphaerae, p_Bacteroidetes, p_Euryarchaeota, p_Patescibacteria, while p_Fusobacteria, p_Firmicutes, and p_Verrucomicrobia were significantly increased compared with the HC group (Figure 3A). At the genus level, we observed 316 significantly different species between ASD and HC groups (Figure 3B). Among them, the top 20 genera that showed a significant increase in the ASD group compared with the HC group included g_Blautia, g_Lachnoclostridium, g_Lachnospira, g_Lachnospiraceae_NK4A136_group, g_Eubacterium_eligens_group, g_Faecalibacterium, g_Dialister, g_Parasutterella, the significantly reduced bacterial flora included g_Prevotella_9, g_Alistipes, g_Agathobacter (Figure 3C). Then analysis at the species level was carried out to find out the bacteria with significant differences, among which the s_Bacteroides_fragilis in the ASD group was significantly increased compared with the HC group, while s_Bacteroides_coprocola_DSM_17136, s_Bacteroides_plebeius, s_Bacteroides_stercoris_ATCC_43183, and the s_Parabacteroides_merdae was significantly reduced (Figure 3D). It showed that indicating that these differential flora may be involved in the gastrointestinal symptomsof ASD.

According to the former study, it demonstrated that the complex microbial ecosystem of the human intestinal tract is unevenly influenced by individualtaxa within different microbial communities (Swiss IBD Cohort Investigators et al., 2019). To characterize potential relationships among bacteria in gut microbial communities, we further constructed co-occurrence networks for each taxon based on significant Spearman correlations. The HC taxa were mainly composed of six co-occurrence networks consisting of dispersed genera from four primary phyla: Proteobacteria, Firmicutes, Actinobacteria and Bacteroidetes, while the ASD taxa were mainly composed of 15 co-occurrence networks consisting of dispersed genera from seven primary phyla: Proteobacteria, Firmicutes, Actinobacteria, Bacteroidetes, Chloroflexi, Acidobacteria, and Patescibacteria (Figure 2C). Two group displayed a co-occurrence network with strong positive correlation among genera. As shown in Figure 2C, the microbial community of the ASD group featured more complicated network. The correlation between the microbiota in the ASD group was distinctly increased compared to that of HC group. To quantify such differences, the average degree of edges (connections) and nodes (genera) were counted in the three microbial networks [ASD (49.78), HC (26.71)]. Taken together, the above analyses further indicating that there may be a disorder in the intestinal microecology of children with ASD.

The linear discriminant analysis (LDA) distribution diagram analysis (LAD score > 3.5) showed a clear alteration of the microbiota characterized by higher p_Frimicutes and p_Actinobacteria levels in ASD individuals (Figure 3E). However, p_Bacteroidetes levels were significantly decreased in ASD group (Figure 3E). The g_Faecalibacterium, g_Blautia, g_Eubacterium_eligens_group, g_Parasutterella, g_Lachnospiraceae_NK4A136_group and g_Veillonella were more abundant in ASD group, while the g_Prevotella 9, g_Agathobacter were more abundant in HC group (Figure 3E). The f_Ruminoccaceae, f_Lachnospiraceae was more abundant in ASD group, while f_Prevotellaceae and f_Enterobacteriaeae was more abundant in HC group (online Supplementary Figure S3). These apparently distinct species could be used as non-invasive biomarkers for the early diagnosis of autism. Taken together, compared with the HC group, the microbial composition of the ASD group showed alterations, further indicating that gut microecology dysbiosis existed in children with ASD.

### Significant differences in metabolic pathways between the ASD group and the HC group

**Figure 4:**
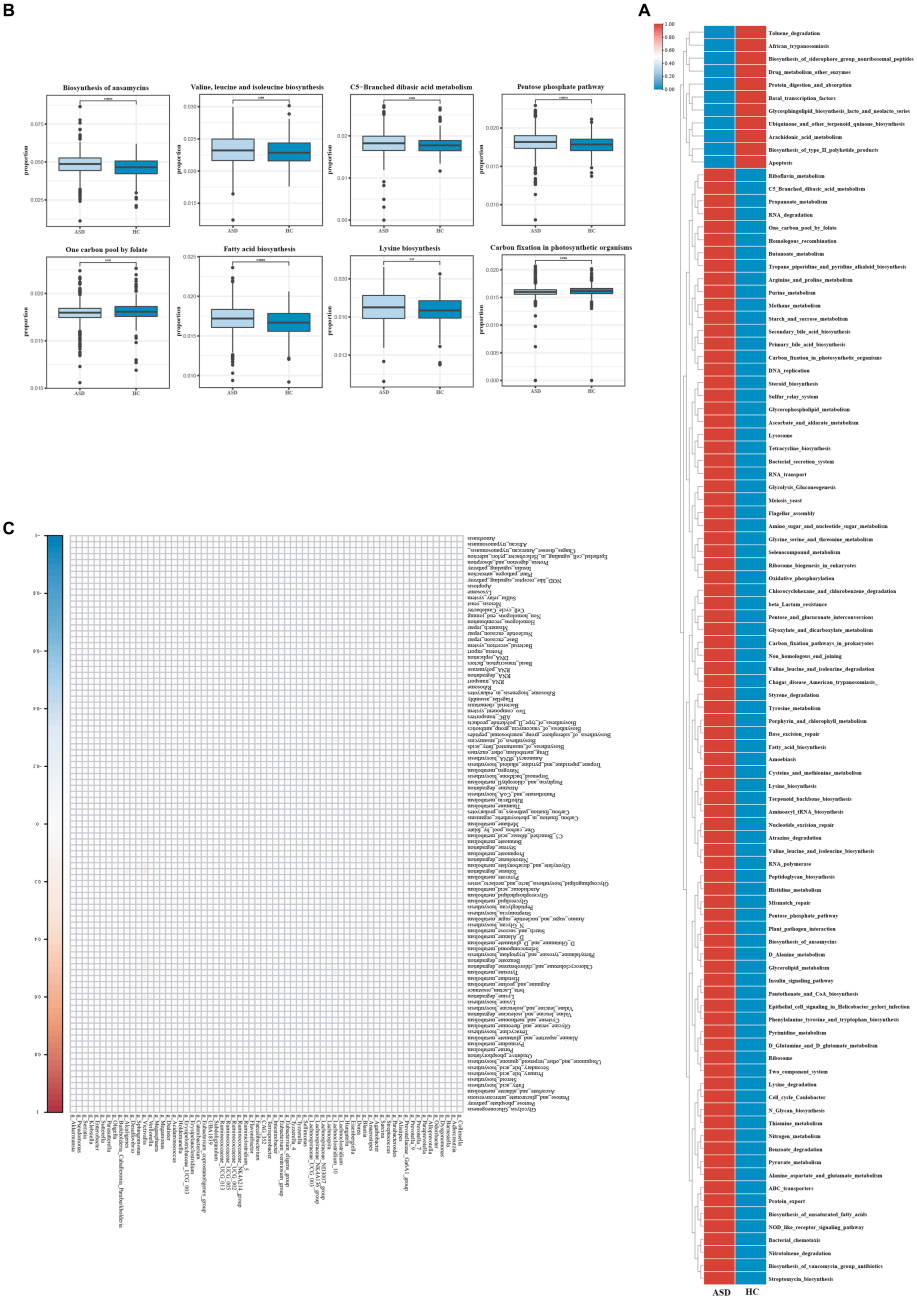
Different metabolic patterns in ASD and HC. **(A)** The average abundance of KEGG pathway differentially enriched in ASD and HC (*p* < 0.05). **(B)** The top 20 of KEGG pathway differentially enriched in ASD and HC (*p* < 0.05). **(C)** The heatmap of correlation between differential gut microbiota and differential metabolites (*p* < 0.05). Red color represents positive correlation, blue color represents negative correlation, and darker color corresponds to a stronger correlation. *p < 0.05, **p < 0.01, and ***p < 0.001.

Ninety-seven Kegg pathways were different between the ASD and HC groups By KEGG pathway analysis (Figure 4A). In particular, among the Top 20 KEGG pathways, One carbon pool By folate pathways, carbon fixation In photosynthetic organisms pathways were significantly less enriched In The ASD group compared with The HC group, while The biosynthesis of ansamycins pathways, valine, leucine and isoleucine biosynthesis pathways, C5 − branched dibasic acid metabolism pathways, pentose phosphate pathway pathways, fatty acid biosynthesis pathways, lysine biosynthesis pathways were significantly more enriched (Figure 4B).

To explore the potential relationships between the gut microbiome changes and metabolic products, a correlation matrix was generated using Spearman correlation (Figure 4C). The abundance of most species such as g_Blautia, g_Prevotella_9, g_Agathobacter, and g_Faecalibacterium, was positively correlated with the level of Penicillin_and_cephalosporin_biosynthesis, Carbon_fixation_in_photosynthetic_organisms, Arachidonic_acid_metabolism, Staphylococcus_aureus_infection. In summary, the abnormal gut microbiota in children with ASD leads to abnormal gut metabolites, which may be the potential reason for the effect of gut microbiota on ASD, although further research was needed to validate the results.

## Discussion

Based on our study, we found significant differences in the diversity, structure, and relative abundance of intestinal microflora between ASD children and healthy children. Therefore, it is crucial to detect the gut microbiota in ASD children for intervention treatment.

Our results showed a significant decrease in gut microbiota diversity in ASD children compared with the HC group (Dan et al., 2020; Wan et al., 2022). These results were consistent with some previous studies (Carissimi et al., 2019; Wang et al., 2019; Dan et al., 2020; Ye et al., 2021). In addition, the Bacteroidetes/Firmicutes ratio of gut microbiota in ASD children was significantly reduced, which was consistent with the observation of gut microbiota in other autistic patients (Tomova et al., 2015; Strati et al., 2017). We found that different bacterial genera, such as g_Blautia, g_Faecalibacterium, g_Lachnospiraceae_NK4A136_group, g_Parasutterella, g_Eubacterium_eligens_group, g_Parasutterella, g_Akkermansia, and g_Anaerostipes significantly increased in the ASD group, while g_Prevotella_9, g_Alistipes, and g_Agathobacter were significantly reduced. These differential gut microbiotas can be used as biomarkers for early screening of ASD. In fact, the research results about ASD gut microbiota are often contradictory (Ho et al., 2020; Iglesias-Vázquez et al., 2020). At the genus level, more controversial findings have been reported, with g_Akkermansia reported to be more abundant in children with ASD (de Angelis et al., 2013; Li et al., 2021), but some studies have shown that the relative abundance of g_Akkermansia is significantly reduced in the gut microbiota of children with ASD (Wang et al., 2011; Zhang et al., 2018). Our data found that the relative abundance of g_Akkermansia in autistic patients was significantly increased in the ASD group. In addition, studies have shown that the relative abundance of g_faecalibacterium in the ASD sleep disorder group was significantly lower compared to healthy controls (Hua et al., 2020), while a meta-analysis reported that g_Faecalibacterium was more abundant in ASD children (Iglesias-Vázquez et al., 2020), consistent with our current research results. Some studies have shown that the Ruminococcaceae family, including Faecalibacterium, Subdoligranulum varia, Clostridium, and Eubacterium, is positively correlated with caproic acid levels (Dan et al., 2020), and ASD is associated with higher levels of caproic acid in the blood (Wang et al., 2012). The acetic acid produced by these intestinal bacteria is a ligand for GPR84, which can enhance the production of pro-inflammatory cytokine IL-12 and its subunit IL-12 P40 induced by lipopolysaccharide (Zhao et al., 2012). It has been reported that IL-12 or IL-12 P40 significantly increased in ASD children (Ashwood et al., 2011; Saghazadeh et al., 2019).

In addition, it was noted that g_Prevotella_9 was depleted in the gut microbiota of ASD children, while g_Blautia was enriched. The results of our g_Blautiade study are contrary to those of previous studies (Xie et al., 2022). However, the decline in Prevotella levels in ASD children was consistent with the results of previous studies (Dan et al., 2020; Zhang et al., 2021). Prevotella was the main bacterial species in the human gut, which could use polysaccharide to produce succinic acid (Kovatcheva-Datchary et al., 2015; Strati et al., 2017). Succinic acid could bind to the succinic acid receptor GPR91 on the surface of dendritic cells to enhance the immune response of antigen-specific T cells and protect the health of the host (Rubic et al., 2008). There is growing evidence that many ASD children have immune dysfunction (Ashwood et al., 2011). Therefore, it was speculated that the immune dysfunction of ASD might be related to the decrease of Prevatella bacteria in the gut, which further affected the physiological and behavioral characteristics of children with ASD. Why do different studies reach different conclusions, which we suspect that it may be due to differences in genetic background, varying manifestations of autism symptoms, as well as differences in age and living environment. Anyhow, these results again indicated that the gut microbiota of children with ASD was significantly different from that of non-ASD individuals.

According to the former study, it showed that Ecological networks of gut microbiota are considered critical for host health and well-being, because it shows that beneficial symbionts and their associated functions are maintained over time (Lozupone et al., 2012; Relman, 2012). In our study, the gut microbial community of children with ASD showed a more complex ecological network where more interactions between bacterial species were observed. It has been shown that highly cooperative microbial communities are perceived as less stable (Coyte and Rakoff-Nahoum, 2019). As previous studies have shown, only gradually increasing the proportion of cooperative interactions within a community almost always reduces the overall rate of return and the likelihood of stability (Coyte et al., 2015). As for key taxa in the network in ASD, Bacteroidetes produce propionic acid and other short-chain fatty acids (SCFA). Rat models administered with propionic acid show increased restrictive/repetitive behavior as well as impaired social behavior (MacFabe et al., 2011). In our study, Bacteroidetes showed the most interactions and identified as key species in the network in children with ASD. The associations between these bacteria and ASD requires further exploration. Besides this, Clostridium genus associated with neurological disorders (Averina et al., 2020) were connected closely with each other under the same genera. Interactions between harmful bacteria may promote and accelerate disease progression (Singer, 2010). Taken together, we observed an unstable and unfavorable ecosystem in the gut microbiome of ASD.

Numerous studies have shown that gut microbiota plays a key role in the biological and physiological characteristics of neurodevelopment, and the establishment of early community relationships among microorganisms may affect the neurodevelopment of children (Dinan and Cryan, 2017; Srikantha and Mohajeri, 2019; Liu et al., 2022). The results showed that, compared with HC group, abnormal microbial function in ASD patients was mainly concentrated in metabolic pathways related to fatty acid, amino acid and aromatic compound metabolism, and partly involved in the metabolism of neurotransmitters. Previous studies have shown that these functions were related to individual nervous system development, neurotransmitter biosynthesis and neuronal response regulation (Needham et al., 2020). For example, abnormalities in the bacterial pathway of tryptophan metabolism have been observed in patients with ASD, and its production of neuroprotective canine and neurotoxic quinolinic acid has been shown to be significantly associated with the severity of ASD (de Angelis et al., 2013; Lou et al., 2022). Our functional analysis of the gut microbiome provided some clues to these potential mechanisms that need to be further investigated in the future.

The differential microbiota and abnormal metabolic pathways identified in our study may have important clinical implications in the early diagnosis and treatment of autism. A large number of previous studies have shown that gut microbiota can be used as a new method for early screening and treatment of autism. Xinyan Xie et al. conducted a case–control study on 101 ASD children and 103 healthy controls in China, and found that Actinobacteria, Proteobacteria, Enterobacteriaceae, and Escherichia were significantly higher in ASD children. However, the numbers of monospheraceae, Blautia and unclassified lachnospiraceae were significantly reduced in ASD children. The gut microbiota of ASD children may have disorders in functional pathways such as amino acid metabolism, coenzyme and vitamin metabolism, and AMPK signaling pathway (Xie et al., 2022). Zhou Dan et al. performed 16S sequencing and metagenomic sequencing analysis on 143 children with clinically diagnosed ASD and 143 age- and sex-matched typically developing (TD) individuals. They found that ASD was associated with alterations in gut microbial profiles and abnormal metabolic activity. ASD patients showed gut ecological imbalance at the phylum, genus and species levels. The findings provide the possibility for future ASD interventions targeting specific microbiota related to neurotransmitter metabolism (Dan et al., 2020). Yingxin Zhao et al. analyzed and compared the gut microbiota of 36 ASD children with gastrointestinal symptoms and 40 TD children. Compared with TD, the gut microbiota of ASD patients with GI symptoms showed a decrease in alpha diversity and butyrate-producing bacteria (such as Faecalibacterium and Coprococcus). The results support the use of gut microbiota as a potential biomarker for early identification of ASD and intervention targeting specific gut microbiota (Zhao et al., 2023).

A strength of this study was the relatively large sample size compared with previous studies on the gut microbiota of ASD children (Supplementary Table S1). However, there were several limitations that need to be addressed in future studies. First, this study was observational only, and longitudinal studies were needed to explore how a disturbed gut microbiome contributes to the development of ASD symptoms. Secondly, due to the availability of data, we were only able to obtain and analyze gut microbiota data, and lacked other data (such as diet, clinical indications, physiology and biochemistry, metabolomics, etc.), which limited the ability of in-depth causal inference. These problems were expected to be solved in the following research.

In summary, our study showed that children with ASD exhibited intestinal microecological disorders at the phylum, genus, and species levels compared with healthy children. These findings supported previous studies on gut microbiota changes in children with ASD and contributed to the development of new approaches for early screening and treatment of ASD based on gut microbiota. However, due to the heterogeneity of ASD phenotypes, further studies in independent populations with larger sample sizes are still needed.

## Data availability statement

The datasets presented in this study can be found in online repositories. The names of the repository/repositories and accession number(s) can be found in the article/Supplementary material.

## Ethics statement

The studies involving humans were approved by his study was approved by the Ethics Committee of Second Affiliated Hospital, Army Medical University (Ethics No. 2023–001-01). Written informed consents were signed by all the children’s legal guardians prior to the study. The clinical trial number: ChiCTR2300074832. The studies were conducted in accordance with the local legislation and institutional requirements. Written informed consent for participation in this study was provided by the participants’ legal guardians/next of kin.

## Author contributions

HL: Conceptualization, Formal analysis, Investigation, Methodology, Project administration, Software, Validation, Writing – original draft, Writing – review & editing. WG: Investigation, Resources, Writing – review & editing. SL: Investigation, Resources, Writing – review & editing. BS: Writing – review & editing. NL: Writing – review & editing. DX: Investigation, Writing – review & editing. ZD: Writing – review & editing. DL: Investigation, Writing – review & editing. WC: Writing – review & editing. WF: Writing – review & editing. JZhe: Writing – review & editing. JZhu: Conceptualization, Funding acquisition, Writing – review & editing.

## Footnotes

^1^ http://www.R-project.org/

^2^ http://huttenhower.sph.harvard.edu/lefse/

^3^ https://www.genome.jp/kegg/pathway.html

## Supplementary material

SUPPLEMENTARY FIGURE S1 Study fowchart.

Supplementary FIGURE S2 Alpha diversity across microbiome samples of ASD and HC.

Supplementary FIGURE S3 Cladograms generated by LEfSe indicating differences in the bacterial taxa between ASD and HC.

Supplementary Table S1 Characteristics of study participants.
