# Supplementary figures and images for: Alteration of the gut microbiota profile in children with autism spectrum disorder in China
Source: Front Microbiol. 2024 Feb 13;14:1326870. doi: 10.3389/fmicb.2023.1326870 (PMC10899803; doi:10.3389/fmicb.2023.1326870)

Autism spectrum disorder

Alteration of Gut Microbiome

Health children

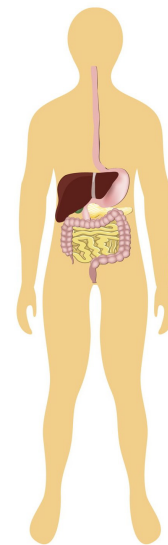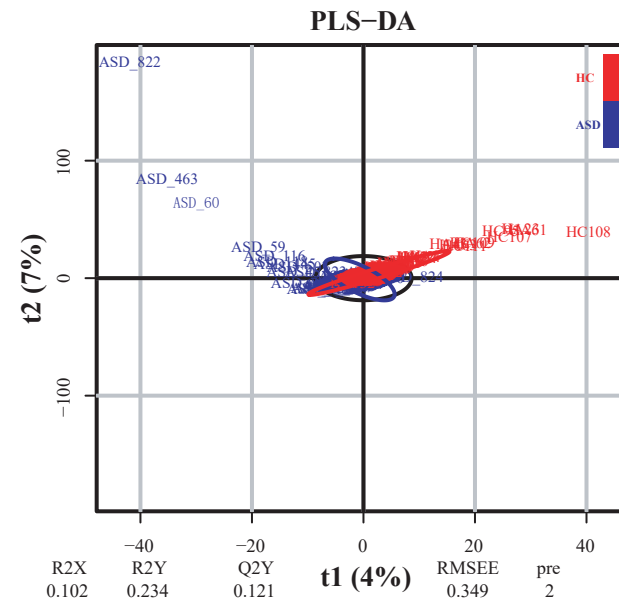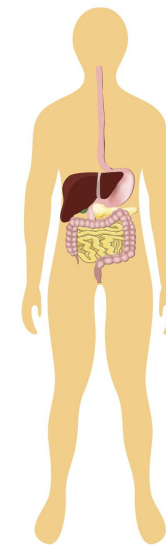

Supplement: SUPPLEMENTARY FIGURE S1 — Study fowchart. [file Data_Sheet_1.PDF]

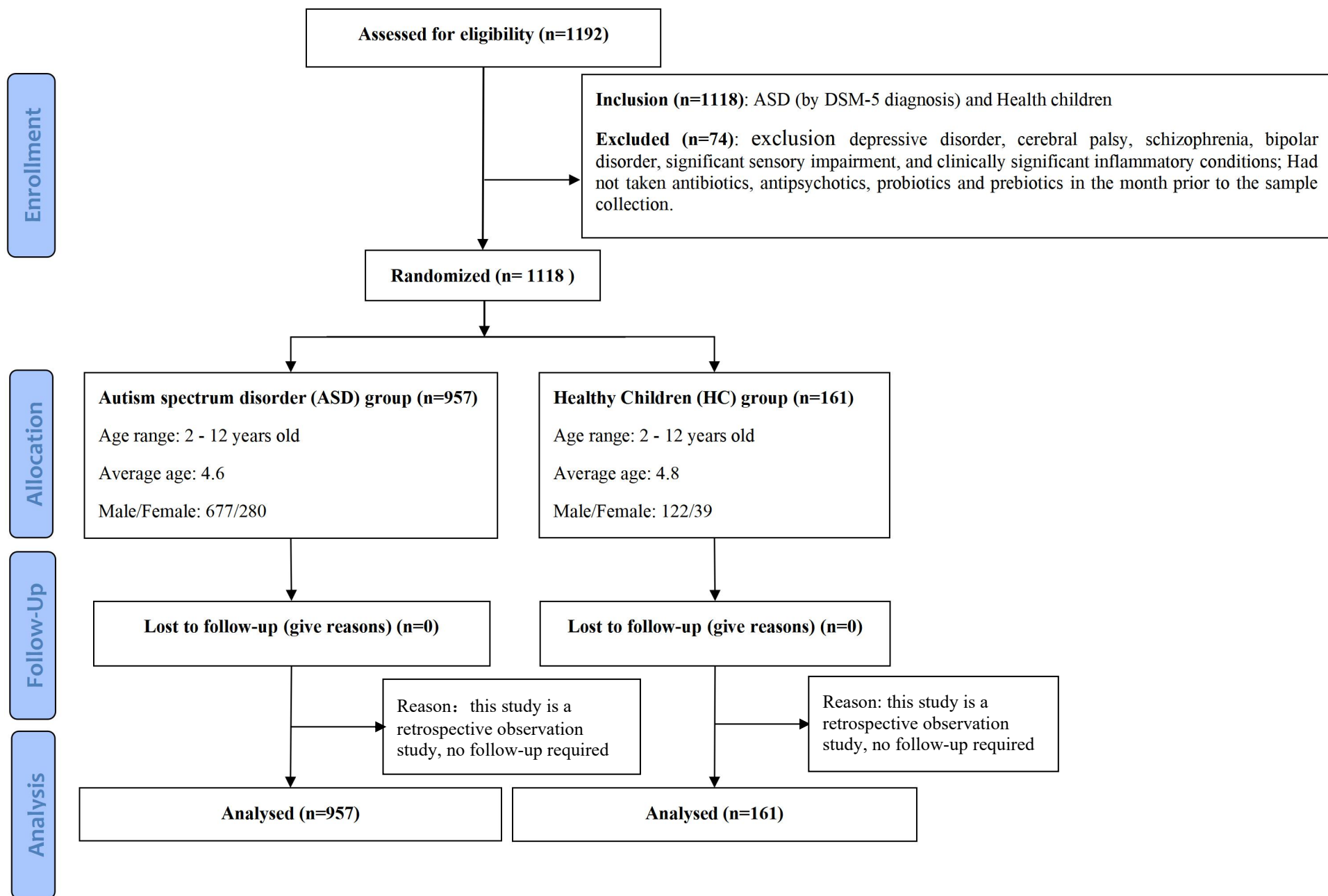

**Figure S1. Study flowchart**

Supplement: Supplementary FIGURE S2 — Alpha diversity across microbiome samples of ASD and HC. [file Data_Sheet_2.PDF]

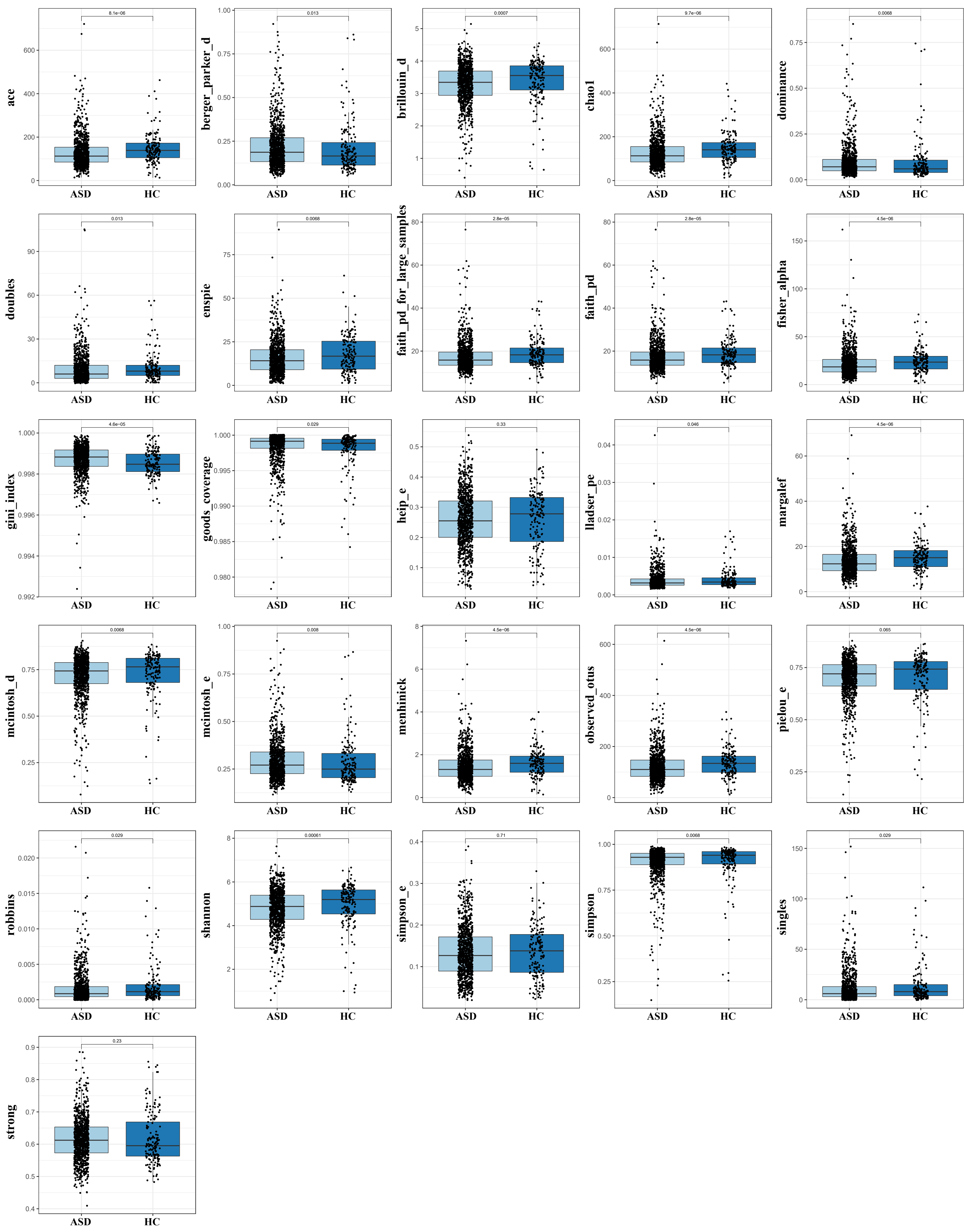

Supplement: Supplementary FIGURE S3 — Cladograms generated by LEfSe indicating differences in the bacterial taxa between ASD and HC. [file Data_Sheet_3.PDF]
