# Supplementary material for: Alteration of the gut microbiota profile in children with autism spectrum disorder in China
Source: Front Microbiol. 2024 Feb 13;14:1326870. doi: 10.3389/fmicb.2023.1326870 (PMC10899803; doi:10.3389/fmicb.2023.1326870)
Supplement: Supplementary file 5 [file Table_1.DOCX]

| Characteristic | ASD | HC | P value |
| --- | --- | --- | --- |
| Total participants (n) | 957 | 161 | ns |
| Age range (years) | 2-12 | 2-12 | ns |
| average age (years) | 4.6 | 4.8 | ns |
| Male/Female | 677/280 | 122/39 | ns |
| Clinical feature | Previous diagnosis of ASD | Community health control | - |

**Table S1. Characteristics of study participants**

ASD, autism spectrum disorders; HC, health children; no significant (ns)

**Participant details table**

| Sample id | Age (year) | Gender | Province | Clinical feature |
| --- | --- | --- | --- | --- |
| ASD_1 | 3 | Female | Liaoning | Previous diagnosis of ASD |
| ASD_2 | 2 | Female | Henan | Previous diagnosis of ASD |
| ASD_3 | 5 | Male | Henan | Previous diagnosis of ASD |
| ASD_4 | 3 | Female | Jilin | Previous diagnosis of ASD |
| ASD_5 | 7 | Male | Shanxi | Previous diagnosis of ASD |
| ASD_6 | 4 | Male | Chongqing | Previous diagnosis of ASD |
| ASD_7 | 8 | Male | Shanxi | Previous diagnosis of ASD |
| ASD_8 | 2 | Male | Hubei | Previous diagnosis of ASD |
| ASD_9 | 4 | Male | Sichuan | Previous diagnosis of ASD |
| ASD_10 | 5 | Male | Shanxi | Previous diagnosis of ASD |
| ASD_11 | 3 | Female | Chongqing | Previous diagnosis of ASD |
| ASD_12 | 3 | Female | Henan | Previous diagnosis of ASD |
| ASD_13 | 12 | Female | Chongqing | Previous diagnosis of ASD |
| ASD_14 | 4 | Male | Jilin | Previous diagnosis of ASD |
| ASD_15 | 2 | Male | Shanxi | Previous diagnosis of ASD |
| ASD_16 | 5 | Female | Henan | Previous diagnosis of ASD |
| ASD_17 | 6 | Female | Shanxi | Previous diagnosis of ASD |
| ASD_18 | 6 | Male | Liaoning | Previous diagnosis of ASD |
| ASD_19 | 3 | Male | Shanxi | Previous diagnosis of ASD |
| ASD_20 | 5 | Male | Shanxi | Previous diagnosis of ASD |
| ASD_21 | 8 | Male | Chongqing | Previous diagnosis of ASD |
| ASD_22 | 3 | Female | Shanxi | Previous diagnosis of ASD |
| ASD_23 | 6 | Female | Chongqing | Previous diagnosis of ASD |
| ASD_24 | 6 | Male | Chongqing | Previous diagnosis of ASD |
| ASD_25 | 4 | Male | Sichuan | Previous diagnosis of ASD |
| ASD_26 | 2 | Male | Fujian | Previous diagnosis of ASD |
| ASD_27 | 3 | Female | Guangxi | Previous diagnosis of ASD |
| ASD_28 | 4 | Female | Chongqing | Previous diagnosis of ASD |
| ASD_29 | 7 | Female | Shanxi | Previous diagnosis of ASD |
| ASD_30 | 4 | Male | Shandong | Previous diagnosis of ASD |
| ASD_31 | 6 | Female | Liaoning | Previous diagnosis of ASD |
| ASD_32 | 4 | Male | Shandong | Previous diagnosis of ASD |
| ASD_33 | 3 | Female | Guangxi | Previous diagnosis of ASD |
| ASD_34 | 12 | Female | Chongqing | Previous diagnosis of ASD |
| ASD_35 | 3 | Female | Liaoning | Previous diagnosis of ASD |
| ASD_36 | 5 | Male | Henan | Previous diagnosis of ASD |
| ASD_37 | 7 | Female | Jilin | Previous diagnosis of ASD |
| ASD_38 | 6 | Male | Shandong | Previous diagnosis of ASD |
| ASD_39 | 3 | Female | Chongqing | Previous diagnosis of ASD |
| ASD_40 | 5 | Male | Guangxi | Previous diagnosis of ASD |
| ASD_41 | 2 | Male | Chongqing | Previous diagnosis of ASD |
| ASD_42 | 6 | Male | Henan | Previous diagnosis of ASD |
| ASD_43 | 5 | Male | Liaoning | Previous diagnosis of ASD |
| ASD_44 | 5 | Male | Fujian | Previous diagnosis of ASD |
| ASD_45 | 2 | Female | Chongqing | Previous diagnosis of ASD |
| ASD_46 | 7 | Male | Chongqing | Previous diagnosis of ASD |
| ASD_47 | 5 | Male | Hubei | Previous diagnosis of ASD |
| ASD_48 | 3 | Male | Chongqing | Previous diagnosis of ASD |
| ASD_49 | 7 | Male | Chongqing | Previous diagnosis of ASD |
| ASD_50 | 3 | Male | Chongqing | Previous diagnosis of ASD |
| ASD_51 | 2 | Male | Hubei | Previous diagnosis of ASD |
| ASD_52 | 3 | Male | Henan | Previous diagnosis of ASD |
| ASD_53 | 3 | Female | Chongqing | Previous diagnosis of ASD |
| ASD_54 | 6 | Male | Shandong | Previous diagnosis of ASD |
| ASD_55 | 3 | Male | Liaoning | Previous diagnosis of ASD |
| ASD_56 | 4 | Male | Henan | Previous diagnosis of ASD |
| ASD_57 | 4 | Male | Shandong | Previous diagnosis of ASD |
| ASD_58 | 2 | Male | Shanxi | Previous diagnosis of ASD |
| ASD_59 | 4 | Male | Chongqing | Previous diagnosis of ASD |
| ASD_60 | 2 | Male | Shandong | Previous diagnosis of ASD |
| ASD_61 | 8 | Female | Heilongjiang | Previous diagnosis of ASD |
| ASD_62 | 4 | Male | Chongqing | Previous diagnosis of ASD |
| ASD_63 | 4 | Male | Guangxi | Previous diagnosis of ASD |
| ASD_64 | 3 | Male | Hubei | Previous diagnosis of ASD |
| ASD_65 | 2 | Male | Chongqing | Previous diagnosis of ASD |
| ASD_66 | 3 | Male | Shanxi | Previous diagnosis of ASD |
| ASD_67 | 7 | Male | Sichuan | Previous diagnosis of ASD |
| ASD_68 | 3 | Female | Henan | Previous diagnosis of ASD |
| ASD_69 | 2 | Male | Guangdong | Previous diagnosis of ASD |
| ASD_70 | 8 | Male | Guangxi | Previous diagnosis of ASD |
| ASD_71 | 7 | Female | Henan | Previous diagnosis of ASD |
| ASD_72 | 3 | Male | Shanxi | Previous diagnosis of ASD |
| ASD_73 | 4 | Female | Chongqing | Previous diagnosis of ASD |
| ASD_74 | 8 | Male | Chongqing | Previous diagnosis of ASD |
| ASD_75 | 5 | Male | Sichuan | Previous diagnosis of ASD |
| ASD_76 | 3 | Male | Chongqing | Previous diagnosis of ASD |
| ASD_77 | 3 | Male | Chongqing | Previous diagnosis of ASD |
| ASD_78 | 3 | Male | Henan | Previous diagnosis of ASD |
| ASD_79 | 3 | Female | Guangxi | Previous diagnosis of ASD |
| ASD_80 | 3 | Male | Chongqing | Previous diagnosis of ASD |
| ASD_81 | 2 | Female | Liaoning | Previous diagnosis of ASD |
| ASD_82 | 3 | Female | Guangdong | Previous diagnosis of ASD |
| ASD_83 | 4 | Male | Chongqing | Previous diagnosis of ASD |
| ASD_84 | 3 | Male | Jilin | Previous diagnosis of ASD |
| ASD_85 | 6 | Female | Chongqing | Previous diagnosis of ASD |
| ASD_86 | 5 | Male | Shandong | Previous diagnosis of ASD |
| ASD_87 | 5 | Male | Sichuan | Previous diagnosis of ASD |
| ASD_88 | 9 | Male | Liaoning | Previous diagnosis of ASD |
| ASD_89 | 4 | Male | Henan | Previous diagnosis of ASD |
| ASD_90 | 4 | Male | Hubei | Previous diagnosis of ASD |
| ASD_91 | 3 | Male | Shanxi | Previous diagnosis of ASD |
| ASD_92 | 4 | Male | Henan | Previous diagnosis of ASD |
| ASD_93 | 3 | Male | Guangdong | Previous diagnosis of ASD |
| ASD_94 | 4 | Male | Guangxi | Previous diagnosis of ASD |
| ASD_95 | 5 | Male | Liaoning | Previous diagnosis of ASD |
| ASD_96 | 7 | Male | Shanxi | Previous diagnosis of ASD |
| ASD_97 | 4 | Male | Guangdong | Previous diagnosis of ASD |
| ASD_98 | 9 | Male | Sichuan | Previous diagnosis of ASD |
| ASD_99 | 5 | Male | Henan | Previous diagnosis of ASD |
| ASD_100 | 4 | Male | Guangdong | Previous diagnosis of ASD |
| ASD_101 | 6 | Male | Henan | Previous diagnosis of ASD |
| ASD_102 | 3 | Male | Shanxi | Previous diagnosis of ASD |
| ASD_103 | 6 | Male | Guangdong | Previous diagnosis of ASD |
| ASD_104 | 3 | Female | Shanxi | Previous diagnosis of ASD |
| ASD_105 | 9 | Male | Jilin | Previous diagnosis of ASD |
| ASD_106 | 4 | Male | Heilongjiang | Previous diagnosis of ASD |
| ASD_107 | 4 | Male | Shanxi | Previous diagnosis of ASD |
| ASD_108 | 2 | Male | Chongqing | Previous diagnosis of ASD |
| ASD_109 | 4 | Female | Chongqing | Previous diagnosis of ASD |
| ASD_110 | 9 | Male | Henan | Previous diagnosis of ASD |
| ASD_111 | 4 | Female | Chongqing | Previous diagnosis of ASD |
| ASD_112 | 5 | Female | Henan | Previous diagnosis of ASD |
| ASD_113 | 11 | Male | Heilongjiang | Previous diagnosis of ASD |
| ASD_114 | 2 | Male | Shandong | Previous diagnosis of ASD |
| ASD_115 | 7 | Male | Shanxi | Previous diagnosis of ASD |
| ASD_116 | 4 | Male | Sichuan | Previous diagnosis of ASD |
| ASD_117 | 4 | Male | Henan | Previous diagnosis of ASD |
| ASD_118 | 4 | Male | Shandong | Previous diagnosis of ASD |
| ASD_119 | 3 | Male | Guangdong | Previous diagnosis of ASD |
| ASD_120 | 4 | Male | Heilongjiang | Previous diagnosis of ASD |
| ASD_121 | 2 | Male | Liaoning | Previous diagnosis of ASD |
| ASD_122 | 4 | Female | Chongqing | Previous diagnosis of ASD |
| ASD_123 | 3 | Male | Shandong | Previous diagnosis of ASD |
| ASD_124 | 3 | Male | Liaoning | Previous diagnosis of ASD |
| ASD_125 | 9 | Female | Liaoning | Previous diagnosis of ASD |
| ASD_126 | 7 | Male | Henan | Previous diagnosis of ASD |
| ASD_127 | 3 | Male | Chongqing | Previous diagnosis of ASD |
| ASD_128 | 4 | Male | Liaoning | Previous diagnosis of ASD |
| ASD_129 | 9 | Male | Henan | Previous diagnosis of ASD |
| ASD_130 | 8 | Male | Fujian | Previous diagnosis of ASD |
| ASD_131 | 3 | Male | Shanxi | Previous diagnosis of ASD |
| ASD_132 | 7 | Male | Chongqing | Previous diagnosis of ASD |
| ASD_133 | 4 | Male | Liaoning | Previous diagnosis of ASD |
| ASD_134 | 3 | Male | Chongqing | Previous diagnosis of ASD |
| ASD_135 | 4 | Male | Chongqing | Previous diagnosis of ASD |
| ASD_136 | 6 | Female | Chongqing | Previous diagnosis of ASD |
| ASD_137 | 3 | Male | Henan | Previous diagnosis of ASD |
| ASD_138 | 3 | Male | Shandong | Previous diagnosis of ASD |
| ASD_139 | 3 | Female | Chongqing | Previous diagnosis of ASD |
| ASD_140 | 5 | Male | Chongqing | Previous diagnosis of ASD |
| ASD_141 | 6 | Female | Guangxi | Previous diagnosis of ASD |
| ASD_142 | 3 | Male | Chongqing | Previous diagnosis of ASD |
| ASD_143 | 3 | Male | Chongqing | Previous diagnosis of ASD |
| ASD_144 | 7 | Female | Chongqing | Previous diagnosis of ASD |
| ASD_145 | 4 | Male | Shandong | Previous diagnosis of ASD |
| ASD_146 | 2 | Female | Shanxi | Previous diagnosis of ASD |
| ASD_147 | 5 | Female | Heilongjiang | Previous diagnosis of ASD |
| ASD_148 | 3 | Female | Heilongjiang | Previous diagnosis of ASD |
| ASD_149 | 5 | Female | Henan | Previous diagnosis of ASD |
| ASD_150 | 6 | Male | Shanxi | Previous diagnosis of ASD |
| ASD_151 | 3 | Male | Heilongjiang | Previous diagnosis of ASD |
| ASD_152 | 5 | Male | Shandong | Previous diagnosis of ASD |
| ASD_153 | 6 | Female | Henan | Previous diagnosis of ASD |
| ASD_154 | 3 | Male | Liaoning | Previous diagnosis of ASD |
| ASD_155 | 5 | Male | Chongqing | Previous diagnosis of ASD |
| ASD_156 | 3 | Male | Henan | Previous diagnosis of ASD |
| ASD_157 | 8 | Male | Sichuan | Previous diagnosis of ASD |
| ASD_158 | 4 | Male | Liaoning | Previous diagnosis of ASD |
| ASD_159 | 3 | Male | Heilongjiang | Previous diagnosis of ASD |
| ASD_160 | 2 | Male | Sichuan | Previous diagnosis of ASD |
| ASD_161 | 3 | Male | Shandong | Previous diagnosis of ASD |
| ASD_162 | 3 | Male | Shandong | Previous diagnosis of ASD |
| ASD_163 | 3 | Male | Fujian | Previous diagnosis of ASD |
| ASD_164 | 3 | Male | Fujian | Previous diagnosis of ASD |
| ASD_165 | 3 | Female | Shanxi | Previous diagnosis of ASD |
| ASD_166 | 6 | Male | Liaoning | Previous diagnosis of ASD |
| ASD_167 | 6 | Male | Guangdong | Previous diagnosis of ASD |
| ASD_168 | 5 | Male | Chongqing | Previous diagnosis of ASD |
| ASD_169 | 4 | Male | Chongqing | Previous diagnosis of ASD |
| ASD_170 | 4 | Male | Shanxi | Previous diagnosis of ASD |
| ASD_171 | 4 | Female | Hubei | Previous diagnosis of ASD |
| ASD_172 | 8 | Female | Guangdong | Previous diagnosis of ASD |
| ASD_173 | 3 | Female | Jilin | Previous diagnosis of ASD |
| ASD_174 | 2 | Male | Liaoning | Previous diagnosis of ASD |
| ASD_175 | 8 | Female | Chongqing | Previous diagnosis of ASD |
| ASD_176 | 3 | Male | Henan | Previous diagnosis of ASD |
| ASD_177 | 2 | Male | Chongqing | Previous diagnosis of ASD |
| ASD_178 | 5 | Male | Chongqing | Previous diagnosis of ASD |
| ASD_179 | 3 | Male | Shanxi | Previous diagnosis of ASD |
| ASD_180 | 5 | Male | Henan | Previous diagnosis of ASD |
| ASD_181 | 4 | Male | Chongqing | Previous diagnosis of ASD |
| ASD_182 | 4 | Male | Fujian | Previous diagnosis of ASD |
| ASD_183 | 4 | Female | Shandong | Previous diagnosis of ASD |
| ASD_184 | 5 | Female | Liaoning | Previous diagnosis of ASD |
| ASD_185 | 4 | Male | Chongqing | Previous diagnosis of ASD |
| ASD_186 | 6 | Male | Shandong | Previous diagnosis of ASD |
| ASD_187 | 3 | Male | Henan | Previous diagnosis of ASD |
| ASD_188 | 9 | Female | Henan | Previous diagnosis of ASD |
| ASD_189 | 3 | Female | Henan | Previous diagnosis of ASD |
| ASD_190 | 4 | Male | Chongqing | Previous diagnosis of ASD |
| ASD_191 | 3 | Female | Hubei | Previous diagnosis of ASD |
| ASD_192 | 4 | Male | Chongqing | Previous diagnosis of ASD |
| ASD_193 | 3 | Female | Chongqing | Previous diagnosis of ASD |
| ASD_194 | 4 | Female | Shanxi | Previous diagnosis of ASD |
| ASD_195 | 2 | Male | Liaoning | Previous diagnosis of ASD |
| ASD_196 | 7 | Male | Chongqing | Previous diagnosis of ASD |
| ASD_197 | 4 | Male | Chongqing | Previous diagnosis of ASD |
| ASD_198 | 3 | Male | Shandong | Previous diagnosis of ASD |
| ASD_199 | 12 | Male | Sichuan | Previous diagnosis of ASD |
| ASD_200 | 9 | Female | Henan | Previous diagnosis of ASD |
| ASD_201 | 8 | Male | Liaoning | Previous diagnosis of ASD |
| ASD_202 | 3 | Male | Shanxi | Previous diagnosis of ASD |
| ASD_203 | 9 | Male | Heilongjiang | Previous diagnosis of ASD |
| ASD_204 | 4 | Male | Chongqing | Previous diagnosis of ASD |
| ASD_205 | 3 | Male | Sichuan | Previous diagnosis of ASD |
| ASD_206 | 6 | Female | Guangxi | Previous diagnosis of ASD |
| ASD_207 | 5 | Male | Chongqing | Previous diagnosis of ASD |
| ASD_208 | 2 | Female | Liaoning | Previous diagnosis of ASD |
| ASD_209 | 4 | Female | Hubei | Previous diagnosis of ASD |
| ASD_210 | 4 | Male | Chongqing | Previous diagnosis of ASD |
| ASD_211 | 5 | Male | Shanxi | Previous diagnosis of ASD |
| ASD_212 | 3 | Male | Chongqing | Previous diagnosis of ASD |
| ASD_213 | 2 | Female | Chongqing | Previous diagnosis of ASD |
| ASD_214 | 4 | Female | Heilongjiang | Previous diagnosis of ASD |
| ASD_215 | 4 | Female | Liaoning | Previous diagnosis of ASD |
| ASD_216 | 4 | Male | Chongqing | Previous diagnosis of ASD |
| ASD_217 | 4 | Male | Guangxi | Previous diagnosis of ASD |
| ASD_218 | 3 | Male | Chongqing | Previous diagnosis of ASD |
| ASD_219 | 3 | Male | Shanxi | Previous diagnosis of ASD |
| ASD_220 | 4 | Female | Guangdong | Previous diagnosis of ASD |
| ASD_221 | 4 | Male | Shandong | Previous diagnosis of ASD |
| ASD_222 | 3 | Female | Shandong | Previous diagnosis of ASD |
| ASD_223 | 6 | Male | Henan | Previous diagnosis of ASD |
| ASD_224 | 5 | Male | Sichuan | Previous diagnosis of ASD |
| ASD_225 | 4 | Male | Henan | Previous diagnosis of ASD |
| ASD_226 | 3 | Male | Liaoning | Previous diagnosis of ASD |
| ASD_227 | 4 | Male | Henan | Previous diagnosis of ASD |
| ASD_228 | 3 | Male | Sichuan | Previous diagnosis of ASD |
| ASD_229 | 5 | Male | Shanxi | Previous diagnosis of ASD |
| ASD_230 | 6 | Female | Henan | Previous diagnosis of ASD |
| ASD_231 | 9 | Male | Chongqing | Previous diagnosis of ASD |
| ASD_232 | 3 | Female | Chongqing | Previous diagnosis of ASD |
| ASD_233 | 2 | Male | Shanxi | Previous diagnosis of ASD |
| ASD_234 | 4 | Male | Hubei | Previous diagnosis of ASD |
| ASD_235 | 5 | Male | Chongqing | Previous diagnosis of ASD |
| ASD_236 | 3 | Male | Jilin | Previous diagnosis of ASD |
| ASD_237 | 4 | Male | Jilin | Previous diagnosis of ASD |
| ASD_238 | 4 | Male | Chongqing | Previous diagnosis of ASD |
| ASD_239 | 9 | Female | Shanxi | Previous diagnosis of ASD |
| ASD_240 | 4 | Male | Shanxi | Previous diagnosis of ASD |
| ASD_241 | 6 | Female | Heilongjiang | Previous diagnosis of ASD |
| ASD_242 | 3 | Male | Sichuan | Previous diagnosis of ASD |
| ASD_243 | 4 | Male | Liaoning | Previous diagnosis of ASD |
| ASD_244 | 3 | Male | Shanxi | Previous diagnosis of ASD |
| ASD_245 | 8 | Female | Sichuan | Previous diagnosis of ASD |
| ASD_246 | 6 | Male | Chongqing | Previous diagnosis of ASD |
| ASD_247 | 3 | Male | Chongqing | Previous diagnosis of ASD |
| ASD_248 | 5 | Male | Shanxi | Previous diagnosis of ASD |
| ASD_249 | 8 | Male | Chongqing | Previous diagnosis of ASD |
| ASD_250 | 5 | Male | Shanxi | Previous diagnosis of ASD |
| ASD_251 | 9 | Male | Liaoning | Previous diagnosis of ASD |
| ASD_252 | 4 | Male | Jilin | Previous diagnosis of ASD |
| ASD_253 | 5 | Male | Henan | Previous diagnosis of ASD |
| ASD_255 | 5 | Male | Henan | Previous diagnosis of ASD |
| ASD_256 | 3 | Female | Liaoning | Previous diagnosis of ASD |
| ASD_257 | 5 | Female | Chongqing | Previous diagnosis of ASD |
| ASD_258 | 12 | Female | Liaoning | Previous diagnosis of ASD |
| ASD_259 | 10 | Male | Chongqing | Previous diagnosis of ASD |
| ASD_260 | 3 | Female | Henan | Previous diagnosis of ASD |
| ASD_261 | 4 | Male | Chongqing | Previous diagnosis of ASD |
| ASD_262 | 4 | Female | Chongqing | Previous diagnosis of ASD |
| ASD_263 | 6 | Male | Henan | Previous diagnosis of ASD |
| ASD_264 | 11 | Male | Sichuan | Previous diagnosis of ASD |
| ASD_265 | 7 | Male | Shanxi | Previous diagnosis of ASD |
| ASD_266 | 3 | Male | Chongqing | Previous diagnosis of ASD |
| ASD_267 | 3 | Female | Heilongjiang | Previous diagnosis of ASD |
| ASD_268 | 3 | Female | Guangxi | Previous diagnosis of ASD |
| ASD_269 | 3 | Female | Fujian | Previous diagnosis of ASD |
| ASD_270 | 7 | Female | Chongqing | Previous diagnosis of ASD |
| ASD_271 | 5 | Male | Sichuan | Previous diagnosis of ASD |
| ASD_272 | 5 | Female | Henan | Previous diagnosis of ASD |
| ASD_273 | 7 | Male | Liaoning | Previous diagnosis of ASD |
| ASD_274 | 10 | Male | Guangxi | Previous diagnosis of ASD |
| ASD_275 | 5 | Male | Guangxi | Previous diagnosis of ASD |
| ASD_276 | 7 | Male | Chongqing | Previous diagnosis of ASD |
| ASD_277 | 9 | Male | Shandong | Previous diagnosis of ASD |
| ASD_278 | 5 | Male | Chongqing | Previous diagnosis of ASD |
| ASD_279 | 3 | Male | Jilin | Previous diagnosis of ASD |
| ASD_280 | 3 | Male | Chongqing | Previous diagnosis of ASD |
| ASD_281 | 4 | Male | Shandong | Previous diagnosis of ASD |
| ASD_282 | 4 | Male | Hubei | Previous diagnosis of ASD |
| ASD_283 | 4 | Female | Chongqing | Previous diagnosis of ASD |
| ASD_284 | 10 | Female | Fujian | Previous diagnosis of ASD |
| ASD_285 | 8 | Male | Heilongjiang | Previous diagnosis of ASD |
| ASD_286 | 7 | Male | Liaoning | Previous diagnosis of ASD |
| ASD_287 | 8 | Male | Sichuan | Previous diagnosis of ASD |
| ASD_288 | 6 | Female | Henan | Previous diagnosis of ASD |
| ASD_289 | 3 | Female | Chongqing | Previous diagnosis of ASD |
| ASD_290 | 12 | Male | Chongqing | Previous diagnosis of ASD |
| ASD_291 | 4 | Male | Guangxi | Previous diagnosis of ASD |
| ASD_292 | 5 | Female | Shanxi | Previous diagnosis of ASD |
| ASD_293 | 3 | Male | Shanxi | Previous diagnosis of ASD |
| ASD_294 | 5 | Male | Fujian | Previous diagnosis of ASD |
| ASD_295 | 3 | Male | Chongqing | Previous diagnosis of ASD |
| ASD_296 | 3 | Male | Shanxi | Previous diagnosis of ASD |
| ASD_297 | 5 | Female | Guangxi | Previous diagnosis of ASD |
| ASD_298 | 3 | Male | Henan | Previous diagnosis of ASD |
| ASD_299 | 6 | Female | Jilin | Previous diagnosis of ASD |
| ASD_300 | 5 | Male | Chongqing | Previous diagnosis of ASD |
| ASD_301 | 4 | Female | Chongqing | Previous diagnosis of ASD |
| ASD_302 | 3 | Male | Shandong | Previous diagnosis of ASD |
| ASD_303 | 8 | Male | Shanxi | Previous diagnosis of ASD |
| ASD_304 | 3 | Male | Sichuan | Previous diagnosis of ASD |
| ASD_305 | 4 | Male | Henan | Previous diagnosis of ASD |
| ASD_306 | 8 | Male | Chongqing | Previous diagnosis of ASD |
| ASD_307 | 4 | Male | Chongqing | Previous diagnosis of ASD |
| ASD_308 | 3 | Male | Chongqing | Previous diagnosis of ASD |
| ASD_309 | 5 | Male | Shanxi | Previous diagnosis of ASD |
| ASD_310 | 4 | Female | Jilin | Previous diagnosis of ASD |
| ASD_311 | 8 | Male | Shanxi | Previous diagnosis of ASD |
| ASD_312 | 3 | Male | Chongqing | Previous diagnosis of ASD |
| ASD_313 | 5 | Male | Sichuan | Previous diagnosis of ASD |
| ASD_314 | 3 | Male | Henan | Previous diagnosis of ASD |
| ASD_315 | 5 | Male | Fujian | Previous diagnosis of ASD |
| ASD_316 | 9 | Male | Henan | Previous diagnosis of ASD |
| ASD_317 | 5 | Male | Chongqing | Previous diagnosis of ASD |
| ASD_318 | 7 | Male | Sichuan | Previous diagnosis of ASD |
| ASD_319 | 9 | Male | Chongqing | Previous diagnosis of ASD |
| ASD_320 | 3 | Female | Fujian | Previous diagnosis of ASD |
| ASD_321 | 4 | Male | Fujian | Previous diagnosis of ASD |
| ASD_322 | 3 | Male | Guangdong | Previous diagnosis of ASD |
| ASD_323 | 5 | Female | Liaoning | Previous diagnosis of ASD |
| ASD_324 | 8 | Male | Chongqing | Previous diagnosis of ASD |
| ASD_325 | 4 | Male | Shandong | Previous diagnosis of ASD |
| ASD_326 | 4 | Male | Shandong | Previous diagnosis of ASD |
| ASD_327 | 4 | Female | Chongqing | Previous diagnosis of ASD |
| ASD_328 | 3 | Male | Chongqing | Previous diagnosis of ASD |
| ASD_329 | 4 | Male | Chongqing | Previous diagnosis of ASD |
| ASD_330 | 4 | Male | Chongqing | Previous diagnosis of ASD |
| ASD_331 | 4 | Female | Chongqing | Previous diagnosis of ASD |
| ASD_332 | 5 | Female | Hubei | Previous diagnosis of ASD |
| ASD_333 | 4 | Male | Sichuan | Previous diagnosis of ASD |
| ASD_334 | 7 | Male | Hubei | Previous diagnosis of ASD |
| ASD_335 | 3 | Female | Hubei | Previous diagnosis of ASD |
| ASD_336 | 8 | Male | Liaoning | Previous diagnosis of ASD |
| ASD_337 | 5 | Male | Henan | Previous diagnosis of ASD |
| ASD_338 | 5 | Male | Chongqing | Previous diagnosis of ASD |
| ASD_339 | 7 | Female | Guangdong | Previous diagnosis of ASD |
| ASD_340 | 4 | Female | Henan | Previous diagnosis of ASD |
| ASD_341 | 4 | Female | Henan | Previous diagnosis of ASD |
| ASD_342 | 2 | Female | Guangxi | Previous diagnosis of ASD |
| ASD_343 | 4 | Male | Chongqing | Previous diagnosis of ASD |
| ASD_344 | 7 | Female | Chongqing | Previous diagnosis of ASD |
| ASD_345 | 5 | Male | Jilin | Previous diagnosis of ASD |
| ASD_346 | 5 | Male | Sichuan | Previous diagnosis of ASD |
| ASD_347 | 3 | Male | Sichuan | Previous diagnosis of ASD |
| ASD_348 | 3 | Male | Guangdong | Previous diagnosis of ASD |
| ASD_349 | 5 | Male | Liaoning | Previous diagnosis of ASD |
| ASD_350 | 4 | Male | Sichuan | Previous diagnosis of ASD |
| ASD_351 | 3 | Male | Liaoning | Previous diagnosis of ASD |
| ASD_352 | 4 | Male | Chongqing | Previous diagnosis of ASD |
| ASD_353 | 3 | Male | Chongqing | Previous diagnosis of ASD |
| ASD_354 | 4 | Male | Liaoning | Previous diagnosis of ASD |
| ASD_355 | 2 | Male | Heilongjiang | Previous diagnosis of ASD |
| ASD_356 | 3 | Female | Chongqing | Previous diagnosis of ASD |
| ASD_357 | 4 | Female | Jilin | Previous diagnosis of ASD |
| ASD_358 | 4 | Female | Chongqing | Previous diagnosis of ASD |
| ASD_359 | 3 | Male | Shandong | Previous diagnosis of ASD |
| ASD_360 | 4 | Male | Chongqing | Previous diagnosis of ASD |
| ASD_361 | 4 | Male | Chongqing | Previous diagnosis of ASD |
| ASD_362 | 3 | Male | Liaoning | Previous diagnosis of ASD |
| ASD_363 | 5 | Male | Shandong | Previous diagnosis of ASD |
| ASD_364 | 4 | Female | Heilongjiang | Previous diagnosis of ASD |
| ASD_365 | 3 | Male | Jilin | Previous diagnosis of ASD |
| ASD_366 | 2 | Female | Sichuan | Previous diagnosis of ASD |
| ASD_367 | 4 | Female | Shandong | Previous diagnosis of ASD |
| ASD_368 | 8 | Female | Chongqing | Previous diagnosis of ASD |
| ASD_369 | 3 | Male | Liaoning | Previous diagnosis of ASD |
| ASD_370 | 4 | Male | Chongqing | Previous diagnosis of ASD |
| ASD_371 | 6 | Female | Chongqing | Previous diagnosis of ASD |
| ASD_372 | 5 | Female | Liaoning | Previous diagnosis of ASD |
| ASD_373 | 3 | Male | Guangdong | Previous diagnosis of ASD |
| ASD_374 | 4 | Female | Chongqing | Previous diagnosis of ASD |
| ASD_375 | 7 | Male | Henan | Previous diagnosis of ASD |
| ASD_376 | 3 | Female | Shanxi | Previous diagnosis of ASD |
| ASD_377 | 3 | Female | Fujian | Previous diagnosis of ASD |
| ASD_378 | 3 | Male | Chongqing | Previous diagnosis of ASD |
| ASD_379 | 4 | Female | Henan | Previous diagnosis of ASD |
| ASD_380 | 5 | Female | Henan | Previous diagnosis of ASD |
| ASD_381 | 3 | Male | Shanxi | Previous diagnosis of ASD |
| ASD_382 | 3 | Male | Hubei | Previous diagnosis of ASD |
| ASD_383 | 5 | Female | Chongqing | Previous diagnosis of ASD |
| ASD_384 | 3 | Female | Hubei | Previous diagnosis of ASD |
| ASD_385 | 4 | Female | Hubei | Previous diagnosis of ASD |
| ASD_386 | 4 | Male | Liaoning | Previous diagnosis of ASD |
| ASD_387 | 3 | Male | Chongqing | Previous diagnosis of ASD |
| ASD_388 | 11 | Female | Shanxi | Previous diagnosis of ASD |
| ASD_389 | 4 | Male | Shanxi | Previous diagnosis of ASD |
| ASD_390 | 3 | Male | Sichuan | Previous diagnosis of ASD |
| ASD_391 | 3 | Male | Chongqing | Previous diagnosis of ASD |
| ASD_392 | 4 | Female | Sichuan | Previous diagnosis of ASD |
| ASD_393 | 3 | Male | Henan | Previous diagnosis of ASD |
| ASD_394 | 6 | Female | Sichuan | Previous diagnosis of ASD |
| ASD_395 | 3 | Male | Liaoning | Previous diagnosis of ASD |
| ASD_396 | 6 | Male | Henan | Previous diagnosis of ASD |
| ASD_397 | 4 | Male | Sichuan | Previous diagnosis of ASD |
| ASD_398 | 3 | Male | Chongqing | Previous diagnosis of ASD |
| ASD_399 | 2 | Male | Shanxi | Previous diagnosis of ASD |
| ASD_400 | 3 | Male | Shanxi | Previous diagnosis of ASD |
| ASD_401 | 4 | Female | Chongqing | Previous diagnosis of ASD |
| ASD_402 | 5 | Male | Fujian | Previous diagnosis of ASD |
| ASD_403 | 5 | Male | Sichuan | Previous diagnosis of ASD |
| ASD_404 | 3 | Male | Jilin | Previous diagnosis of ASD |
| ASD_405 | 5 | Female | Chongqing | Previous diagnosis of ASD |
| ASD_406 | 4 | Male | Henan | Previous diagnosis of ASD |
| ASD_407 | 3 | Male | Henan | Previous diagnosis of ASD |
| ASD_408 | 2 | Male | Sichuan | Previous diagnosis of ASD |
| ASD_409 | 8 | Male | Jilin | Previous diagnosis of ASD |
| ASD_410 | 5 | Male | Chongqing | Previous diagnosis of ASD |
| ASD_411 | 7 | Male | Guangdong | Previous diagnosis of ASD |
| ASD_412 | 9 | Male | Hubei | Previous diagnosis of ASD |
| ASD_413 | 4 | Female | Hubei | Previous diagnosis of ASD |
| ASD_414 | 5 | Male | Chongqing | Previous diagnosis of ASD |
| ASD_415 | 8 | Female | Liaoning | Previous diagnosis of ASD |
| ASD_416 | 2 | Male | Shanxi | Previous diagnosis of ASD |
| ASD_417 | 5 | Female | Guangxi | Previous diagnosis of ASD |
| ASD_418 | 6 | Female | Henan | Previous diagnosis of ASD |
| ASD_419 | 2 | Male | Shandong | Previous diagnosis of ASD |
| ASD_420 | 3 | Male | Liaoning | Previous diagnosis of ASD |
| ASD_421 | 3 | Male | Fujian | Previous diagnosis of ASD |
| ASD_422 | 6 | Male | Fujian | Previous diagnosis of ASD |
| ASD_423 | 5 | Male | Sichuan | Previous diagnosis of ASD |
| ASD_424 | 4 | Male | Jilin | Previous diagnosis of ASD |
| ASD_425 | 3 | Male | Jilin | Previous diagnosis of ASD |
| ASD_426 | 2 | Male | Guangdong | Previous diagnosis of ASD |
| ASD_427 | 2 | Male | Hubei | Previous diagnosis of ASD |
| ASD_428 | 10 | Male | Shanxi | Previous diagnosis of ASD |
| ASD_429 | 6 | Male | Chongqing | Previous diagnosis of ASD |
| ASD_430 | 5 | Male | Chongqing | Previous diagnosis of ASD |
| ASD_431 | 3 | Male | Chongqing | Previous diagnosis of ASD |
| ASD_432 | 6 | Male | Liaoning | Previous diagnosis of ASD |
| ASD_433 | 4 | Male | Henan | Previous diagnosis of ASD |
| ASD_434 | 3 | Female | Chongqing | Previous diagnosis of ASD |
| ASD_435 | 4 | Female | Chongqing | Previous diagnosis of ASD |
| ASD_436 | 4 | Male | Chongqing | Previous diagnosis of ASD |
| ASD_437 | 4 | Male | Shanxi | Previous diagnosis of ASD |
| ASD_438 | 5 | Male | Liaoning | Previous diagnosis of ASD |
| ASD_439 | 10 | Male | Shandong | Previous diagnosis of ASD |
| ASD_440 | 4 | Male | Sichuan | Previous diagnosis of ASD |
| ASD_441 | 3 | Male | Chongqing | Previous diagnosis of ASD |
| ASD_442 | 4 | Female | Heilongjiang | Previous diagnosis of ASD |
| ASD_443 | 4 | Male | Jilin | Previous diagnosis of ASD |
| ASD_444 | 4 | Female | Guangxi | Previous diagnosis of ASD |
| ASD_445 | 4 | Male | Hubei | Previous diagnosis of ASD |
| ASD_446 | 3 | Male | Shandong | Previous diagnosis of ASD |
| ASD_447 | 2 | Male | Guangdong | Previous diagnosis of ASD |
| ASD_448 | 7 | Male | Guangdong | Previous diagnosis of ASD |
| ASD_449 | 3 | Male | Sichuan | Previous diagnosis of ASD |
| ASD_450 | 3 | Male | Chongqing | Previous diagnosis of ASD |
| ASD_451 | 8 | Male | Chongqing | Previous diagnosis of ASD |
| ASD_452 | 3 | Female | Fujian | Previous diagnosis of ASD |
| ASD_453 | 10 | Male | Fujian | Previous diagnosis of ASD |
| ASD_454 | 3 | Male | Henan | Previous diagnosis of ASD |
| ASD_455 | 2 | Female | Liaoning | Previous diagnosis of ASD |
| ASD_456 | 4 | Male | Shandong | Previous diagnosis of ASD |
| ASD_457 | 8 | Male | Henan | Previous diagnosis of ASD |
| ASD_458 | 5 | Female | Henan | Previous diagnosis of ASD |
| ASD_459 | 5 | Male | Sichuan | Previous diagnosis of ASD |
| ASD_460 | 7 | Male | Guangdong | Previous diagnosis of ASD |
| ASD_461 | 4 | Male | Shanxi | Previous diagnosis of ASD |
| ASD_462 | 9 | Male | Henan | Previous diagnosis of ASD |
| ASD_463 | 3 | Male | Chongqing | Previous diagnosis of ASD |
| ASD_464 | 7 | Male | Chongqing | Previous diagnosis of ASD |
| ASD_465 | 8 | Female | Hubei | Previous diagnosis of ASD |
| ASD_466 | 5 | Male | Henan | Previous diagnosis of ASD |
| ASD_467 | 6 | Male | Chongqing | Previous diagnosis of ASD |
| ASD_468 | 2 | Female | Fujian | Previous diagnosis of ASD |
| ASD_469 | 3 | Male | Heilongjiang | Previous diagnosis of ASD |
| ASD_470 | 3 | Male | Guangxi | Previous diagnosis of ASD |
| ASD_471 | 2 | Male | Shandong | Previous diagnosis of ASD |
| ASD_472 | 4 | Male | Chongqing | Previous diagnosis of ASD |
| ASD_473 | 5 | Male | Chongqing | Previous diagnosis of ASD |
| ASD_474 | 3 | Male | Sichuan | Previous diagnosis of ASD |
| ASD_475 | 12 | Male | Henan | Previous diagnosis of ASD |
| ASD_476 | 2 | Male | Henan | Previous diagnosis of ASD |
| ASD_477 | 3 | Male | Shanxi | Previous diagnosis of ASD |
| ASD_478 | 3 | Male | Chongqing | Previous diagnosis of ASD |
| ASD_479 | 4 | Male | Shandong | Previous diagnosis of ASD |
| ASD_480 | 3 | Male | Henan | Previous diagnosis of ASD |
| ASD_481 | 3 | Male | Liaoning | Previous diagnosis of ASD |
| ASD_482 | 6 | Male | Chongqing | Previous diagnosis of ASD |
| ASD_483 | 2 | Male | Chongqing | Previous diagnosis of ASD |
| ASD_484 | 5 | Male | Shanxi | Previous diagnosis of ASD |
| ASD_485 | 5 | Female | Shanxi | Previous diagnosis of ASD |
| ASD_486 | 3 | Male | Chongqing | Previous diagnosis of ASD |
| ASD_487 | 3 | Female | Chongqing | Previous diagnosis of ASD |
| ASD_488 | 3 | Male | Henan | Previous diagnosis of ASD |
| ASD_489 | 5 | Male | Chongqing | Previous diagnosis of ASD |
| ASD_490 | 8 | Female | Jilin | Previous diagnosis of ASD |
| ASD_491 | 5 | Female | Henan | Previous diagnosis of ASD |
| ASD_492 | 4 | Male | Chongqing | Previous diagnosis of ASD |
| ASD_493 | 4 | Male | Shanxi | Previous diagnosis of ASD |
| ASD_494 | 5 | Female | Shanxi | Previous diagnosis of ASD |
| ASD_495 | 3 | Male | Chongqing | Previous diagnosis of ASD |
| ASD_496 | 6 | Male | Jilin | Previous diagnosis of ASD |
| ASD_497 | 3 | Female | Henan | Previous diagnosis of ASD |
| ASD_498 | 3 | Female | Hubei | Previous diagnosis of ASD |
| ASD_499 | 3 | Male | Heilongjiang | Previous diagnosis of ASD |
| ASD_500 | 5 | Female | Sichuan | Previous diagnosis of ASD |
| ASD_501 | 3 | Male | Sichuan | Previous diagnosis of ASD |
| ASD_502 | 9 | Female | Guangxi | Previous diagnosis of ASD |
| ASD_503 | 2 | Male | Shanxi | Previous diagnosis of ASD |
| ASD_504 | 4 | Male | Shanxi | Previous diagnosis of ASD |
| ASD_505 | 4 | Male | Chongqing | Previous diagnosis of ASD |
| ASD_506 | 8 | Female | Jilin | Previous diagnosis of ASD |
| ASD_507 | 7 | Male | Liaoning | Previous diagnosis of ASD |
| ASD_508 | 9 | Female | Liaoning | Previous diagnosis of ASD |
| ASD_509 | 10 | Female | Shanxi | Previous diagnosis of ASD |
| ASD_510 | 8 | Male | Shandong | Previous diagnosis of ASD |
| ASD_511 | 4 | Male | Guangxi | Previous diagnosis of ASD |
| ASD_512 | 12 | Male | Chongqing | Previous diagnosis of ASD |
| ASD_513 | 3 | Female | Shandong | Previous diagnosis of ASD |
| ASD_514 | 7 | Female | Heilongjiang | Previous diagnosis of ASD |
| ASD_515 | 5 | Male | Sichuan | Previous diagnosis of ASD |
| ASD_516 | 6 | Female | Chongqing | Previous diagnosis of ASD |
| ASD_517 | 3 | Female | Jilin | Previous diagnosis of ASD |
| ASD_518 | 4 | Male | Hubei | Previous diagnosis of ASD |
| ASD_519 | 3 | Female | Heilongjiang | Previous diagnosis of ASD |
| ASD_520 | 3 | Male | Jilin | Previous diagnosis of ASD |
| ASD_521 | 3 | Male | Heilongjiang | Previous diagnosis of ASD |
| ASD_522 | 4 | Male | Liaoning | Previous diagnosis of ASD |
| ASD_523 | 3 | Female | Shanxi | Previous diagnosis of ASD |
| ASD_524 | 6 | Male | Henan | Previous diagnosis of ASD |
| ASD_525 | 6 | Male | Chongqing | Previous diagnosis of ASD |
| ASD_526 | 3 | Male | Liaoning | Previous diagnosis of ASD |
| ASD_527 | 3 | Male | Guangxi | Previous diagnosis of ASD |
| ASD_528 | 2 | Male | Chongqing | Previous diagnosis of ASD |
| ASD_529 | 5 | Male | Shandong | Previous diagnosis of ASD |
| ASD_530 | 8 | Male | Shandong | Previous diagnosis of ASD |
| ASD_531 | 6 | Male | Chongqing | Previous diagnosis of ASD |
| ASD_532 | 6 | Male | Liaoning | Previous diagnosis of ASD |
| ASD_533 | 6 | Male | Jilin | Previous diagnosis of ASD |
| ASD_534 | 4 | Female | Heilongjiang | Previous diagnosis of ASD |
| ASD_535 | 3 | Male | Chongqing | Previous diagnosis of ASD |
| ASD_536 | 5 | Female | Shanxi | Previous diagnosis of ASD |
| ASD_537 | 4 | Male | Shandong | Previous diagnosis of ASD |
| ASD_538 | 3 | Female | Hubei | Previous diagnosis of ASD |
| ASD_539 | 12 | Male | Guangdong | Previous diagnosis of ASD |
| ASD_540 | 5 | Male | Sichuan | Previous diagnosis of ASD |
| ASD_541 | 4 | Male | Hubei | Previous diagnosis of ASD |
| ASD_542 | 6 | Male | Shanxi | Previous diagnosis of ASD |
| ASD_543 | 9 | Male | Guangxi | Previous diagnosis of ASD |
| ASD_544 | 4 | Female | Chongqing | Previous diagnosis of ASD |
| ASD_545 | 2 | Female | Shandong | Previous diagnosis of ASD |
| ASD_546 | 10 | Female | Guangxi | Previous diagnosis of ASD |
| ASD_547 | 3 | Male | Heilongjiang | Previous diagnosis of ASD |
| ASD_548 | 3 | Male | Henan | Previous diagnosis of ASD |
| ASD_549 | 4 | Female | Chongqing | Previous diagnosis of ASD |
| ASD_550 | 6 | Male | Chongqing | Previous diagnosis of ASD |
| ASD_551 | 7 | Male | Heilongjiang | Previous diagnosis of ASD |
| ASD_552 | 5 | Male | Sichuan | Previous diagnosis of ASD |
| ASD_553 | 3 | Male | Sichuan | Previous diagnosis of ASD |
| ASD_554 | 3 | Male | Liaoning | Previous diagnosis of ASD |
| ASD_555 | 3 | Male | Shanxi | Previous diagnosis of ASD |
| ASD_556 | 8 | Male | Sichuan | Previous diagnosis of ASD |
| ASD_557 | 4 | Male | Chongqing | Previous diagnosis of ASD |
| ASD_558 | 4 | Female | Chongqing | Previous diagnosis of ASD |
| ASD_559 | 4 | Male | Chongqing | Previous diagnosis of ASD |
| ASD_560 | 7 | Male | Chongqing | Previous diagnosis of ASD |
| ASD_561 | 3 | Male | Heilongjiang | Previous diagnosis of ASD |
| ASD_562 | 6 | Male | Liaoning | Previous diagnosis of ASD |
| ASD_563 | 3 | Male | Sichuan | Previous diagnosis of ASD |
| ASD_564 | 6 | Female | Liaoning | Previous diagnosis of ASD |
| ASD_565 | 2 | Male | Henan | Previous diagnosis of ASD |
| ASD_566 | 4 | Male | Guangdong | Previous diagnosis of ASD |
| ASD_567 | 5 | Male | Chongqing | Previous diagnosis of ASD |
| ASD_568 | 9 | Male | Chongqing | Previous diagnosis of ASD |
| ASD_569 | 2 | Male | Shandong | Previous diagnosis of ASD |
| ASD_570 | 5 | Female | Henan | Previous diagnosis of ASD |
| ASD_571 | 3 | Male | Shanxi | Previous diagnosis of ASD |
| ASD_572 | 4 | Female | Guangdong | Previous diagnosis of ASD |
| ASD_573 | 3 | Male | Guangdong | Previous diagnosis of ASD |
| ASD_574 | 5 | Male | Chongqing | Previous diagnosis of ASD |
| ASD_575 | 3 | Female | Sichuan | Previous diagnosis of ASD |
| ASD_576 | 9 | Male | Henan | Previous diagnosis of ASD |
| ASD_577 | 7 | Female | Liaoning | Previous diagnosis of ASD |
| ASD_578 | 3 | Male | Shandong | Previous diagnosis of ASD |
| ASD_579 | 3 | Male | Liaoning | Previous diagnosis of ASD |
| ASD_580 | 4 | Female | Henan | Previous diagnosis of ASD |
| ASD_581 | 6 | Male | Hubei | Previous diagnosis of ASD |
| ASD_582 | 4 | Female | Shandong | Previous diagnosis of ASD |
| ASD_583 | 3 | Female | Shandong | Previous diagnosis of ASD |
| ASD_584 | 7 | Male | Shanxi | Previous diagnosis of ASD |
| ASD_585 | 3 | Male | Chongqing | Previous diagnosis of ASD |
| ASD_586 | 3 | Female | Sichuan | Previous diagnosis of ASD |
| ASD_587 | 2 | Male | Shanxi | Previous diagnosis of ASD |
| ASD_588 | 5 | Female | Shanxi | Previous diagnosis of ASD |
| ASD_589 | 3 | Female | Fujian | Previous diagnosis of ASD |
| ASD_590 | 4 | Female | Jilin | Previous diagnosis of ASD |
| ASD_591 | 3 | Male | Chongqing | Previous diagnosis of ASD |
| ASD_592 | 3 | Male | Shanxi | Previous diagnosis of ASD |
| ASD_593 | 4 | Male | Fujian | Previous diagnosis of ASD |
| ASD_594 | 3 | Male | Shanxi | Previous diagnosis of ASD |
| ASD_595 | 6 | Male | Chongqing | Previous diagnosis of ASD |
| ASD_596 | 5 | Male | Guangdong | Previous diagnosis of ASD |
| ASD_597 | 4 | Male | Guangdong | Previous diagnosis of ASD |
| ASD_598 | 3 | Male | Liaoning | Previous diagnosis of ASD |
| ASD_599 | 7 | Female | Henan | Previous diagnosis of ASD |
| ASD_600 | 3 | Female | Liaoning | Previous diagnosis of ASD |
| ASD_601 | 8 | Male | Shandong | Previous diagnosis of ASD |
| ASD_602 | 4 | Male | Shandong | Previous diagnosis of ASD |
| ASD_603 | 3 | Female | Chongqing | Previous diagnosis of ASD |
| ASD_604 | 3 | Male | Liaoning | Previous diagnosis of ASD |
| ASD_605 | 5 | Female | Chongqing | Previous diagnosis of ASD |
| ASD_606 | 3 | Female | Chongqing | Previous diagnosis of ASD |
| ASD_607 | 9 | Female | Shanxi | Previous diagnosis of ASD |
| ASD_608 | 9 | Male | Guangdong | Previous diagnosis of ASD |
| ASD_609 | 3 | Male | Liaoning | Previous diagnosis of ASD |
| ASD_610 | 7 | Male | Henan | Previous diagnosis of ASD |
| ASD_611 | 3 | Female | Guangxi | Previous diagnosis of ASD |
| ASD_612 | 3 | Male | Chongqing | Previous diagnosis of ASD |
| ASD_613 | 5 | Male | Henan | Previous diagnosis of ASD |
| ASD_614 | 3 | Male | Guangxi | Previous diagnosis of ASD |
| ASD_615 | 4 | Male | Fujian | Previous diagnosis of ASD |
| ASD_616 | 3 | Male | Henan | Previous diagnosis of ASD |
| ASD_617 | 6 | Male | Shandong | Previous diagnosis of ASD |
| ASD_618 | 3 | Male | Fujian | Previous diagnosis of ASD |
| ASD_619 | 5 | Female | Chongqing | Previous diagnosis of ASD |
| ASD_620 | 11 | Male | Shandong | Previous diagnosis of ASD |
| ASD_621 | 6 | Male | Shanxi | Previous diagnosis of ASD |
| ASD_622 | 3 | Female | Sichuan | Previous diagnosis of ASD |
| ASD_623 | 3 | Male | Chongqing | Previous diagnosis of ASD |
| ASD_624 | 4 | Female | Shanxi | Previous diagnosis of ASD |
| ASD_625 | 3 | Female | Henan | Previous diagnosis of ASD |
| ASD_626 | 2 | Male | Heilongjiang | Previous diagnosis of ASD |
| ASD_627 | 3 | Male | Henan | Previous diagnosis of ASD |
| ASD_628 | 5 | Male | Heilongjiang | Previous diagnosis of ASD |
| ASD_629 | 3 | Female | Shanxi | Previous diagnosis of ASD |
| ASD_630 | 4 | Female | Shanxi | Previous diagnosis of ASD |
| ASD_631 | 3 | Male | Chongqing | Previous diagnosis of ASD |
| ASD_632 | 4 | Female | Chongqing | Previous diagnosis of ASD |
| ASD_633 | 3 | Male | Chongqing | Previous diagnosis of ASD |
| ASD_634 | 3 | Male | Shanxi | Previous diagnosis of ASD |
| ASD_635 | 8 | Male | Chongqing | Previous diagnosis of ASD |
| ASD_636 | 5 | Male | Chongqing | Previous diagnosis of ASD |
| ASD_637 | 8 | Female | Henan | Previous diagnosis of ASD |
| ASD_638 | 5 | Male | Shanxi | Previous diagnosis of ASD |
| ASD_639 | 3 | Male | Shandong | Previous diagnosis of ASD |
| ASD_640 | 6 | Male | Heilongjiang | Previous diagnosis of ASD |
| ASD_641 | 4 | Female | Shanxi | Previous diagnosis of ASD |
| ASD_642 | 4 | Male | Guangdong | Previous diagnosis of ASD |
| ASD_643 | 7 | Male | Shanxi | Previous diagnosis of ASD |
| ASD_644 | 4 | Male | Hubei | Previous diagnosis of ASD |
| ASD_645 | 5 | Male | Heilongjiang | Previous diagnosis of ASD |
| ASD_646 | 3 | Male | Guangdong | Previous diagnosis of ASD |
| ASD_647 | 4 | Male | Guangdong | Previous diagnosis of ASD |
| ASD_648 | 3 | Male | Shandong | Previous diagnosis of ASD |
| ASD_649 | 6 | Male | Shanxi | Previous diagnosis of ASD |
| ASD_650 | 6 | Male | Sichuan | Previous diagnosis of ASD |
| ASD_651 | 9 | Male | Sichuan | Previous diagnosis of ASD |
| ASD_652 | 12 | Male | Shanxi | Previous diagnosis of ASD |
| ASD_653 | 3 | Female | Shanxi | Previous diagnosis of ASD |
| ASD_654 | 3 | Male | Sichuan | Previous diagnosis of ASD |
| ASD_655 | 7 | Male | Sichuan | Previous diagnosis of ASD |
| ASD_656 | 11 | Male | Shandong | Previous diagnosis of ASD |
| ASD_657 | 4 | Male | Jilin | Previous diagnosis of ASD |
| ASD_658 | 5 | Male | Henan | Previous diagnosis of ASD |
| ASD_659 | 3 | Male | Chongqing | Previous diagnosis of ASD |
| ASD_660 | 4 | Male | Fujian | Previous diagnosis of ASD |
| ASD_661 | 6 | Male | Chongqing | Previous diagnosis of ASD |
| ASD_662 | 3 | Male | Shanxi | Previous diagnosis of ASD |
| ASD_663 | 3 | Male | Chongqing | Previous diagnosis of ASD |
| ASD_664 | 3 | Male | Hubei | Previous diagnosis of ASD |
| ASD_665 | 5 | Male | Fujian | Previous diagnosis of ASD |
| ASD_666 | 3 | Male | Shanxi | Previous diagnosis of ASD |
| ASD_667 | 8 | Male | Heilongjiang | Previous diagnosis of ASD |
| ASD_668 | 5 | Male | Chongqing | Previous diagnosis of ASD |
| ASD_669 | 3 | Male | Henan | Previous diagnosis of ASD |
| ASD_670 | 4 | Female | Sichuan | Previous diagnosis of ASD |
| ASD_671 | 8 | Female | Chongqing | Previous diagnosis of ASD |
| ASD_672 | 3 | Male | Chongqing | Previous diagnosis of ASD |
| ASD_673 | 3 | Male | Henan | Previous diagnosis of ASD |
| ASD_674 | 5 | Male | Shanxi | Previous diagnosis of ASD |
| ASD_675 | 12 | Male | Chongqing | Previous diagnosis of ASD |
| ASD_676 | 6 | Female | Chongqing | Previous diagnosis of ASD |
| ASD_677 | 5 | Male | Shanxi | Previous diagnosis of ASD |
| ASD_678 | 9 | Male | Guangdong | Previous diagnosis of ASD |
| ASD_679 | 5 | Male | Shandong | Previous diagnosis of ASD |
| ASD_680 | 3 | Male | Jilin | Previous diagnosis of ASD |
| ASD_681 | 2 | Male | Chongqing | Previous diagnosis of ASD |
| ASD_682 | 4 | Male | Guangdong | Previous diagnosis of ASD |
| ASD_683 | 4 | Male | Chongqing | Previous diagnosis of ASD |
| ASD_684 | 4 | Male | Chongqing | Previous diagnosis of ASD |
| ASD_685 | 3 | Male | Shandong | Previous diagnosis of ASD |
| ASD_686 | 2 | Female | Shandong | Previous diagnosis of ASD |
| ASD_687 | 8 | Male | Hubei | Previous diagnosis of ASD |
| ASD_688 | 6 | Female | Hubei | Previous diagnosis of ASD |
| ASD_689 | 4 | Male | Fujian | Previous diagnosis of ASD |
| ASD_690 | 4 | Male | Jilin | Previous diagnosis of ASD |
| ASD_691 | 3 | Male | Guangdong | Previous diagnosis of ASD |
| ASD_692 | 9 | Male | Henan | Previous diagnosis of ASD |
| ASD_693 | 3 | Male | Guangxi | Previous diagnosis of ASD |
| ASD_694 | 4 | Male | Shanxi | Previous diagnosis of ASD |
| ASD_695 | 3 | Male | Henan | Previous diagnosis of ASD |
| ASD_696 | 4 | Male | Henan | Previous diagnosis of ASD |
| ASD_697 | 5 | Female | Chongqing | Previous diagnosis of ASD |
| ASD_698 | 3 | Male | Liaoning | Previous diagnosis of ASD |
| ASD_699 | 5 | Male | Chongqing | Previous diagnosis of ASD |
| ASD_700 | 8 | Female | Guangdong | Previous diagnosis of ASD |
| ASD_701 | 3 | Male | Henan | Previous diagnosis of ASD |
| ASD_702 | 6 | Female | Sichuan | Previous diagnosis of ASD |
| ASD_703 | 6 | Female | Chongqing | Previous diagnosis of ASD |
| ASD_704 | 3 | Male | Liaoning | Previous diagnosis of ASD |
| ASD_705 | 5 | Male | Chongqing | Previous diagnosis of ASD |
| ASD_706 | 3 | Female | Heilongjiang | Previous diagnosis of ASD |
| ASD_707 | 8 | Male | Sichuan | Previous diagnosis of ASD |
| ASD_708 | 4 | Male | Sichuan | Previous diagnosis of ASD |
| ASD_709 | 3 | Female | Chongqing | Previous diagnosis of ASD |
| ASD_710 | 9 | Male | Liaoning | Previous diagnosis of ASD |
| ASD_711 | 3 | Male | Chongqing | Previous diagnosis of ASD |
| ASD_712 | 3 | Male | Shanxi | Previous diagnosis of ASD |
| ASD_713 | 5 | Male | Shanxi | Previous diagnosis of ASD |
| ASD_714 | 4 | Male | Heilongjiang | Previous diagnosis of ASD |
| ASD_715 | 4 | Female | Liaoning | Previous diagnosis of ASD |
| ASD_716 | 4 | Male | Henan | Previous diagnosis of ASD |
| ASD_717 | 3 | Male | Guangdong | Previous diagnosis of ASD |
| ASD_718 | 2 | Male | Fujian | Previous diagnosis of ASD |
| ASD_719 | 5 | Female | Henan | Previous diagnosis of ASD |
| ASD_720 | 2 | Male | Chongqing | Previous diagnosis of ASD |
| ASD_721 | 6 | Male | Shandong | Previous diagnosis of ASD |
| ASD_722 | 4 | Female | Guangdong | Previous diagnosis of ASD |
| ASD_723 | 2 | Male | Shanxi | Previous diagnosis of ASD |
| ASD_724 | 5 | Female | Shandong | Previous diagnosis of ASD |
| ASD_725 | 3 | Male | Sichuan | Previous diagnosis of ASD |
| ASD_726 | 9 | Male | Shandong | Previous diagnosis of ASD |
| ASD_727 | 2 | Male | Henan | Previous diagnosis of ASD |
| ASD_728 | 4 | Male | Guangxi | Previous diagnosis of ASD |
| ASD_729 | 5 | Female | Henan | Previous diagnosis of ASD |
| ASD_730 | 4 | Male | Jilin | Previous diagnosis of ASD |
| ASD_731 | 7 | Male | Fujian | Previous diagnosis of ASD |
| ASD_732 | 2 | Male | Chongqing | Previous diagnosis of ASD |
| ASD_733 | 3 | Male | Henan | Previous diagnosis of ASD |
| ASD_734 | 10 | Male | Shanxi | Previous diagnosis of ASD |
| ASD_735 | 4 | Male | Heilongjiang | Previous diagnosis of ASD |
| ASD_736 | 3 | Male | Hubei | Previous diagnosis of ASD |
| ASD_737 | 3 | Female | Guangxi | Previous diagnosis of ASD |
| ASD_738 | 7 | Female | Guangxi | Previous diagnosis of ASD |
| ASD_739 | 2 | Female | Chongqing | Previous diagnosis of ASD |
| ASD_740 | 5 | Male | Heilongjiang | Previous diagnosis of ASD |
| ASD_741 | 4 | Female | Chongqing | Previous diagnosis of ASD |
| ASD_742 | 6 | Female | Hubei | Previous diagnosis of ASD |
| ASD_743 | 4 | Male | Shanxi | Previous diagnosis of ASD |
| ASD_744 | 5 | Male | Chongqing | Previous diagnosis of ASD |
| ASD_745 | 8 | Male | Chongqing | Previous diagnosis of ASD |
| ASD_746 | 2 | Male | Guangxi | Previous diagnosis of ASD |
| ASD_747 | 4 | Male | Shandong | Previous diagnosis of ASD |
| ASD_748 | 5 | Male | Chongqing | Previous diagnosis of ASD |
| ASD_749 | 3 | Female | Sichuan | Previous diagnosis of ASD |
| ASD_750 | 3 | Male | Sichuan | Previous diagnosis of ASD |
| ASD_751 | 4 | Female | Jilin | Previous diagnosis of ASD |
| ASD_752 | 3 | Male | Shanxi | Previous diagnosis of ASD |
| ASD_753 | 3 | Male | Heilongjiang | Previous diagnosis of ASD |
| ASD_754 | 8 | Female | Jilin | Previous diagnosis of ASD |
| ASD_755 | 3 | Female | Fujian | Previous diagnosis of ASD |
| ASD_756 | 2 | Male | Henan | Previous diagnosis of ASD |
| ASD_757 | 3 | Female | Shanxi | Previous diagnosis of ASD |
| ASD_758 | 4 | Female | Shanxi | Previous diagnosis of ASD |
| ASD_759 | 4 | Female | Chongqing | Previous diagnosis of ASD |
| ASD_760 | 5 | Male | Liaoning | Previous diagnosis of ASD |
| ASD_761 | 4 | Male | Chongqing | Previous diagnosis of ASD |
| ASD_762 | 3 | Male | Shanxi | Previous diagnosis of ASD |
| ASD_763 | 4 | Male | Hubei | Previous diagnosis of ASD |
| ASD_764 | 5 | Female | Chongqing | Previous diagnosis of ASD |
| ASD_765 | 2 | Male | Guangdong | Previous diagnosis of ASD |
| ASD_766 | 2 | Male | Shandong | Previous diagnosis of ASD |
| ASD_767 | 5 | Female | Chongqing | Previous diagnosis of ASD |
| ASD_768 | 2 | Female | Chongqing | Previous diagnosis of ASD |
| ASD_769 | 3 | Male | Liaoning | Previous diagnosis of ASD |
| ASD_770 | 9 | Male | Henan | Previous diagnosis of ASD |
| ASD_771 | 2 | Male | Sichuan | Previous diagnosis of ASD |
| ASD_772 | 6 | Male | Chongqing | Previous diagnosis of ASD |
| ASD_773 | 4 | Female | Chongqing | Previous diagnosis of ASD |
| ASD_774 | 3 | Male | Guangxi | Previous diagnosis of ASD |
| ASD_775 | 2 | Male | Henan | Previous diagnosis of ASD |
| ASD_776 | 5 | Male | Guangdong | Previous diagnosis of ASD |
| ASD_777 | 3 | Male | Liaoning | Previous diagnosis of ASD |
| ASD_778 | 8 | Female | Sichuan | Previous diagnosis of ASD |
| ASD_779 | 4 | Female | Sichuan | Previous diagnosis of ASD |
| ASD_780 | 4 | Male | Henan | Previous diagnosis of ASD |
| ASD_781 | 12 | Female | Henan | Previous diagnosis of ASD |
| ASD_782 | 3 | Male | Shanxi | Previous diagnosis of ASD |
| ASD_783 | 4 | Female | Henan | Previous diagnosis of ASD |
| ASD_784 | 5 | Male | Guangxi | Previous diagnosis of ASD |
| ASD_785 | 5 | Male | Sichuan | Previous diagnosis of ASD |
| ASD_786 | 11 | Male | Henan | Previous diagnosis of ASD |
| ASD_787 | 2 | Female | Shandong | Previous diagnosis of ASD |
| ASD_788 | 4 | Male | Guangdong | Previous diagnosis of ASD |
| ASD_789 | 3 | Male | Henan | Previous diagnosis of ASD |
| ASD_790 | 6 | Male | Shanxi | Previous diagnosis of ASD |
| ASD_791 | 6 | Male | Guangdong | Previous diagnosis of ASD |
| ASD_792 | 4 | Male | Jilin | Previous diagnosis of ASD |
| ASD_793 | 5 | Female | Hubei | Previous diagnosis of ASD |
| ASD_794 | 9 | Male | Liaoning | Previous diagnosis of ASD |
| ASD_796 | 3 | Female | Sichuan | Previous diagnosis of ASD |
| ASD_797 | 3 | Male | Shanxi | Previous diagnosis of ASD |
| ASD_798 | 3 | Female | Heilongjiang | Previous diagnosis of ASD |
| ASD_799 | 4 | Female | Liaoning | Previous diagnosis of ASD |
| ASD_800 | 4 | Male | Shanxi | Previous diagnosis of ASD |
| ASD_801 | 5 | Male | Liaoning | Previous diagnosis of ASD |
| ASD_802 | 5 | Male | Fujian | Previous diagnosis of ASD |
| ASD_803 | 5 | Female | Heilongjiang | Previous diagnosis of ASD |
| ASD_804 | 4 | Female | Henan | Previous diagnosis of ASD |
| ASD_805 | 5 | Male | Chongqing | Previous diagnosis of ASD |
| ASD_806 | 4 | Female | Liaoning | Previous diagnosis of ASD |
| ASD_807 | 3 | Male | Liaoning | Previous diagnosis of ASD |
| ASD_808 | 5 | Female | Chongqing | Previous diagnosis of ASD |
| ASD_809 | 3 | Male | Henan | Previous diagnosis of ASD |
| ASD_810 | 11 | Male | Chongqing | Previous diagnosis of ASD |
| ASD_811 | 11 | Male | Jilin | Previous diagnosis of ASD |
| ASD_812 | 5 | Male | Shanxi | Previous diagnosis of ASD |
| ASD_813 | 5 | Female | Shandong | Previous diagnosis of ASD |
| ASD_814 | 3 | Female | Shandong | Previous diagnosis of ASD |
| ASD_815 | 6 | Female | Fujian | Previous diagnosis of ASD |
| ASD_816 | 6 | Male | Chongqing | Previous diagnosis of ASD |
| ASD_817 | 12 | Male | Jilin | Previous diagnosis of ASD |
| ASD_818 | 4 | Male | Fujian | Previous diagnosis of ASD |
| ASD_819 | 5 | Male | Sichuan | Previous diagnosis of ASD |
| ASD_820 | 5 | Male | Fujian | Previous diagnosis of ASD |
| ASD_821 | 4 | Male | Shanxi | Previous diagnosis of ASD |
| ASD_822 | 3 | Male | Henan | Previous diagnosis of ASD |
| ASD_823 | 6 | Male | Sichuan | Previous diagnosis of ASD |
| ASD_824 | 3 | Male | Chongqing | Previous diagnosis of ASD |
| ASD_825 | 5 | Female | Jilin | Previous diagnosis of ASD |
| ASD_827 | 4 | Female | Shandong | Previous diagnosis of ASD |
| ASD_828 | 4 | Male | Chongqing | Previous diagnosis of ASD |
| ASD_829 | 3 | Male | Shanxi | Previous diagnosis of ASD |
| ASD_830 | 4 | Female | Shanxi | Previous diagnosis of ASD |
| ASD_831 | 6 | Female | Chongqing | Previous diagnosis of ASD |
| ASD_832 | 6 | Male | Sichuan | Previous diagnosis of ASD |
| ASD_833 | 5 | Female | Chongqing | Previous diagnosis of ASD |
| ASD_834 | 11 | Male | Fujian | Previous diagnosis of ASD |
| ASD_835 | 3 | Male | Guangxi | Previous diagnosis of ASD |
| ASD_836 | 6 | Male | Chongqing | Previous diagnosis of ASD |
| ASD_837 | 5 | Male | Shandong | Previous diagnosis of ASD |
| ASD_838 | 4 | Male | Liaoning | Previous diagnosis of ASD |
| ASD_839 | 5 | Male | Liaoning | Previous diagnosis of ASD |
| ASD_840 | 4 | Male | Sichuan | Previous diagnosis of ASD |
| ASD_841 | 3 | Male | Shanxi | Previous diagnosis of ASD |
| ASD_842 | 5 | Male | Shanxi | Previous diagnosis of ASD |
| ASD_843 | 3 | Male | Chongqing | Previous diagnosis of ASD |
| ASD_844 | 3 | Female | Chongqing | Previous diagnosis of ASD |
| ASD_845 | 7 | Male | Shandong | Previous diagnosis of ASD |
| ASD_846 | 8 | Male | Chongqing | Previous diagnosis of ASD |
| ASD_847 | 6 | Male | Shanxi | Previous diagnosis of ASD |
| ASD_848 | 3 | Female | Heilongjiang | Previous diagnosis of ASD |
| ASD_849 | 4 | Male | Chongqing | Previous diagnosis of ASD |
| ASD_850 | 4 | Female | Sichuan | Previous diagnosis of ASD |
| ASD_851 | 5 | Female | Sichuan | Previous diagnosis of ASD |
| ASD_852 | 4 | Male | Liaoning | Previous diagnosis of ASD |
| ASD_853 | 5 | Male | Shanxi | Previous diagnosis of ASD |
| ASD_854 | 4 | Male | Shandong | Previous diagnosis of ASD |
| ASD_855 | 2 | Female | Jilin | Previous diagnosis of ASD |
| ASD_856 | 8 | Male | Chongqing | Previous diagnosis of ASD |
| ASD_857 | 4 | Male | Liaoning | Previous diagnosis of ASD |
| ASD_858 | 7 | Female | Hubei | Previous diagnosis of ASD |
| ASD_859 | 3 | Male | Henan | Previous diagnosis of ASD |
| ASD_860 | 10 | Male | Liaoning | Previous diagnosis of ASD |
| ASD_861 | 6 | Male | Chongqing | Previous diagnosis of ASD |
| ASD_862 | 3 | Male | Chongqing | Previous diagnosis of ASD |
| ASD_863 | 4 | Male | Shanxi | Previous diagnosis of ASD |
| ASD_864 | 3 | Male | Chongqing | Previous diagnosis of ASD |
| ASD_865 | 4 | Male | Shandong | Previous diagnosis of ASD |
| ASD_866 | 4 | Male | Henan | Previous diagnosis of ASD |
| ASD_867 | 3 | Male | Shandong | Previous diagnosis of ASD |
| ASD_868 | 4 | Female | Shandong | Previous diagnosis of ASD |
| ASD_869 | 4 | Male | Fujian | Previous diagnosis of ASD |
| ASD_870 | 2 | Male | Sichuan | Previous diagnosis of ASD |
| ASD_871 | 3 | Male | Chongqing | Previous diagnosis of ASD |
| ASD_872 | 3 | Female | Hubei | Previous diagnosis of ASD |
| ASD_873 | 3 | Male | Jilin | Previous diagnosis of ASD |
| ASD_874 | 6 | Female | Liaoning | Previous diagnosis of ASD |
| ASD_875 | 10 | Female | Henan | Previous diagnosis of ASD |
| ASD_876 | 3 | Male | Chongqing | Previous diagnosis of ASD |
| ASD_877 | 3 | Male | Hubei | Previous diagnosis of ASD |
| ASD_878 | 4 | Male | Sichuan | Previous diagnosis of ASD |
| ASD_879 | 4 | Male | Sichuan | Previous diagnosis of ASD |
| ASD_880 | 4 | Female | Shandong | Previous diagnosis of ASD |
| ASD_881 | 5 | Male | Shandong | Previous diagnosis of ASD |
| ASD_882 | 5 | Male | Shanxi | Previous diagnosis of ASD |
| ASD_883 | 3 | Female | Sichuan | Previous diagnosis of ASD |
| ASD_884 | 3 | Male | Sichuan | Previous diagnosis of ASD |
| ASD_885 | 3 | Male | Shandong | Previous diagnosis of ASD |
| ASD_886 | 4 | Male | Chongqing | Previous diagnosis of ASD |
| ASD_887 | 4 | Female | Henan | Previous diagnosis of ASD |
| ASD_888 | 3 | Female | Liaoning | Previous diagnosis of ASD |
| ASD_889 | 8 | Female | Shanxi | Previous diagnosis of ASD |
| ASD_890 | 3 | Male | Heilongjiang | Previous diagnosis of ASD |
| ASD_891 | 4 | Male | Shanxi | Previous diagnosis of ASD |
| ASD_892 | 4 | Female | Henan | Previous diagnosis of ASD |
| ASD_893 | 6 | Male | Sichuan | Previous diagnosis of ASD |
| ASD_894 | 3 | Female | Henan | Previous diagnosis of ASD |
| ASD_895 | 12 | Female | Chongqing | Previous diagnosis of ASD |
| ASD_896 | 3 | Male | Henan | Previous diagnosis of ASD |
| ASD_897 | 5 | Female | Chongqing | Previous diagnosis of ASD |
| ASD_898 | 4 | Male | Fujian | Previous diagnosis of ASD |
| ASD_899 | 4 | Female | Sichuan | Previous diagnosis of ASD |
| ASD_900 | 5 | Female | Fujian | Previous diagnosis of ASD |
| ASD_901 | 9 | Male | Shandong | Previous diagnosis of ASD |
| ASD_902 | 3 | Male | Jilin | Previous diagnosis of ASD |
| ASD_903 | 8 | Male | Henan | Previous diagnosis of ASD |
| ASD_904 | 4 | Male | Guangdong | Previous diagnosis of ASD |
| ASD_905 | 12 | Male | Henan | Previous diagnosis of ASD |
| ASD_906 | 4 | Female | Shandong | Previous diagnosis of ASD |
| ASD_907 | 4 | Male | Shanxi | Previous diagnosis of ASD |
| ASD_908 | 3 | Male | Henan | Previous diagnosis of ASD |
| ASD_909 | 4 | Male | Henan | Previous diagnosis of ASD |
| ASD_910 | 4 | Male | Shanxi | Previous diagnosis of ASD |
| ASD_911 | 3 | Male | Chongqing | Previous diagnosis of ASD |
| ASD_912 | 3 | Male | Chongqing | Previous diagnosis of ASD |
| ASD_913 | 6 | Female | Shandong | Previous diagnosis of ASD |
| ASD_914 | 3 | Male | Chongqing | Previous diagnosis of ASD |
| ASD_915 | 3 | Male | Shanxi | Previous diagnosis of ASD |
| ASD_916 | 3 | Male | Chongqing | Previous diagnosis of ASD |
| ASD_917 | 5 | Male | Shandong | Previous diagnosis of ASD |
| ASD_918 | 2 | Male | Chongqing | Previous diagnosis of ASD |
| ASD_919 | 7 | Male | Chongqing | Previous diagnosis of ASD |
| ASD_920 | 2 | Female | Hubei | Previous diagnosis of ASD |
| ASD_921 | 3 | Male | Guangxi | Previous diagnosis of ASD |
| ASD_922 | 12 | Male | Chongqing | Previous diagnosis of ASD |
| ASD_923 | 12 | Male | Heilongjiang | Previous diagnosis of ASD |
| ASD_924 | 4 | Female | Chongqing | Previous diagnosis of ASD |
| ASD_925 | 3 | Male | Guangxi | Previous diagnosis of ASD |
| ASD_926 | 5 | Male | Guangxi | Previous diagnosis of ASD |
| ASD_927 | 3 | Male | Chongqing | Previous diagnosis of ASD |
| ASD_928 | 4 | Female | Guangxi | Previous diagnosis of ASD |
| ASD_929 | 4 | Female | Henan | Previous diagnosis of ASD |
| ASD_930 | 5 | Male | Chongqing | Previous diagnosis of ASD |
| ASD_931 | 5 | Female | Shandong | Previous diagnosis of ASD |
| ASD_932 | 2 | Male | Henan | Previous diagnosis of ASD |
| ASD_933 | 6 | Female | Chongqing | Previous diagnosis of ASD |
| ASD_934 | 3 | Male | Guangxi | Previous diagnosis of ASD |
| ASD_935 | 8 | Male | Shanxi | Previous diagnosis of ASD |
| ASD_936 | 4 | Male | Sichuan | Previous diagnosis of ASD |
| ASD_937 | 3 | Male | Liaoning | Previous diagnosis of ASD |
| ASD_938 | 7 | Female | Shandong | Previous diagnosis of ASD |
| ASD_939 | 3 | Male | Hubei | Previous diagnosis of ASD |
| ASD_940 | 5 | Female | Shandong | Previous diagnosis of ASD |
| ASD_941 | 8 | Male | Chongqing | Previous diagnosis of ASD |
| ASD_942 | 8 | Male | Henan | Previous diagnosis of ASD |
| ASD_943 | 3 | Male | Chongqing | Previous diagnosis of ASD |
| ASD_944 | 4 | Female | Fujian | Previous diagnosis of ASD |
| ASD_945 | 5 | Male | Chongqing | Previous diagnosis of ASD |
| ASD_946 | 4 | Male | Sichuan | Previous diagnosis of ASD |
| ASD_947 | 3 | Male | Fujian | Previous diagnosis of ASD |
| ASD_948 | 4 | Female | Chongqing | Previous diagnosis of ASD |
| ASD_949 | 4 | Female | Henan | Previous diagnosis of ASD |
| ASD_950 | 9 | Male | Chongqing | Previous diagnosis of ASD |
| ASD_951 | 3 | Male | Shandong | Previous diagnosis of ASD |
| ASD_952 | 4 | Male | Guangxi | Previous diagnosis of ASD |
| ASD_953 | 3 | Male | Chongqing | Previous diagnosis of ASD |
| ASD_954 | 4 | Male | Shanxi | Previous diagnosis of ASD |
| ASD_955 | 3 | Male | Henan | Previous diagnosis of ASD |
| ASD_956 | 3 | Male | Shanxi | Previous diagnosis of ASD |
| ASD_957 | 6 | Female | Henan | Previous diagnosis of ASD |
| ASD_958 | 5 | Male | Sichuan | Previous diagnosis of ASD |
| ASD_959 | 3 | Female | Shanxi | Previous diagnosis of ASD |
| ASD_960 | 3 | Female | Chongqing | Previous diagnosis of ASD |
| HC1 | 4 | Male | Chongqing | Community health control |
| HC2 | 4 | Female | Chongqing | Community health control |
| HC3 | 5 | Male | Chongqing | Community health control |
| HC4 | 5 | Male | Chongqing | Community health control |
| HC5 | 5 | Male | Chongqing | Community health control |
| HC6 | 4 | Male | Chongqing | Community health control |
| HC7 | 5 | Male | Chongqing | Community health control |
| HC8 | 4 | Male | Chongqing | Community health control |
| HC9 | 3 | Male | Chongqing | Community health control |
| HC10 | 5 | Male | Chongqing | Community health control |
| HC11 | 3 | Male | Chongqing | Community health control |
| HC12 | 11 | Male | Chongqing | Community health control |
| HC13 | 11 | Male | Chongqing | Community health control |
| HC14 | 5 | Male | Chongqing | Community health control |
| HC15 | 5 | Male | Chongqing | Community health control |
| HC16 | 3 | Male | Chongqing | Community health control |
| HC17 | 6 | Female | Chongqing | Community health control |
| HC18 | 6 | Female | Chongqing | Community health control |
| HC19 | 12 | Male | Chongqing | Community health control |
| HC20 | 4 | Male | Chongqing | Community health control |
| HC21 | 5 | Male | Chongqing | Community health control |
| HC22 | 5 | Male | Chongqing | Community health control |
| HC23 | 4 | Male | Chongqing | Community health control |
| HC24 | 3 | Male | Chongqing | Community health control |
| HC25 | 11 | Male | Chongqing | Community health control |
| HC26 | 3 | Male | Chongqing | Community health control |
| HC27 | 5 | Male | Chongqing | Community health control |
| HC28 | 4 | Female | Chongqing | Community health control |
| HC29 | 4 | Male | Chongqing | Community health control |
| HC30 | 3 | Female | Chongqing | Community health control |
| HC31 | 4 | Male | Chongqing | Community health control |
| HC32 | 6 | Male | Chongqing | Community health control |
| HC33 | 6 | Male | Chongqing | Community health control |
| HC34 | 5 | Male | Chongqing | Community health control |
| HC35 | 11 | Male | Chongqing | Community health control |
| HC36 | 3 | Male | Chongqing | Community health control |
| HC37 | 6 | Male | Chongqing | Community health control |
| HC38 | 5 | Male | Chongqing | Community health control |
| HC39 | 4 | Male | Chongqing | Community health control |
| HC40 | 5 | Male | Chongqing | Community health control |
| HC41 | 4 | Female | Chongqing | Community health control |
| HC42 | 3 | Male | Chongqing | Community health control |
| HC43 | 5 | Male | Chongqing | Community health control |
| HC44 | 3 | Male | Chongqing | Community health control |
| HC45 | 3 | Female | Chongqing | Community health control |
| HC46 | 7 | Male | Chongqing | Community health control |
| HC47 | 8 | Female | Chongqing | Community health control |
| HC48 | 6 | Male | Chongqing | Community health control |
| HC49 | 3 | Female | Chongqing | Community health control |
| HC50 | 4 | Male | Chongqing | Community health control |
| HC51 | 4 | Male | Chongqing | Community health control |
| HC52 | 5 | Female | Chongqing | Community health control |
| HC53 | 4 | Male | Chongqing | Community health control |
| HC54 | 5 | Female | Chongqing | Community health control |
| HC55 | 4 | Male | Chongqing | Community health control |
| HC56 | 2 | Male | Chongqing | Community health control |
| HC57 | 8 | Male | Chongqing | Community health control |
| HC58 | 4 | Female | Chongqing | Community health control |
| HC59 | 7 | Male | Chongqing | Community health control |
| HC60 | 3 | Male | Chongqing | Community health control |
| HC61 | 10 | Male | Chongqing | Community health control |
| HC62 | 6 | Male | Chongqing | Community health control |
| HC63 | 3 | Male | Chongqing | Community health control |
| HC64 | 4 | Male | Chongqing | Community health control |
| HC65 | 3 | Male | Chongqing | Community health control |
| HC66 | 4 | Male | Chongqing | Community health control |
| HC67 | 4 | Male | Chongqing | Community health control |
| HC68 | 3 | Male | Chongqing | Community health control |
| HC69 | 4 | Male | Chongqing | Community health control |
| HC70 | 4 | Male | Chongqing | Community health control |
| HC71 | 2 | Male | Chongqing | Community health control |
| HC72 | 3 | Female | Chongqing | Community health control |
| HC73 | 3 | Male | Chongqing | Community health control |
| HC74 | 3 | Male | Chongqing | Community health control |
| HC75 | 6 | Male | Chongqing | Community health control |
| HC76 | 10 | Male | Chongqing | Community health control |
| HC77 | 3 | Female | Chongqing | Community health control |
| HC78 | 3 | Female | Chongqing | Community health control |
| HC79 | 4 | Female | Chongqing | Community health control |
| HC80 | 4 | Male | Chongqing | Community health control |
| HC81 | 4 | Male | Chongqing | Community health control |
| HC82 | 5 | Male | Chongqing | Community health control |
| HC83 | 5 | Male | Chongqing | Community health control |
| HC84 | 3 | Female | Chongqing | Community health control |
| HC85 | 3 | Female | Chongqing | Community health control |
| HC86 | 3 | Male | Chongqing | Community health control |
| HC87 | 4 | Male | Chongqing | Community health control |
| HC88 | 4 | Female | Chongqing | Community health control |
| HC89 | 3 | Male | Chongqing | Community health control |
| HC90 | 8 | Female | Chongqing | Community health control |
| HC91 | 3 | Male | Chongqing | Community health control |
| HC92 | 4 | Female | Chongqing | Community health control |
| HC93 | 4 | Male | Chongqing | Community health control |
| HC94 | 6 | Male | Chongqing | Community health control |
| HC95 | 3 | Male | Chongqing | Community health control |
| HC96 | 12 | Male | Chongqing | Community health control |
| HC97 | 3 | Female | Chongqing | Community health control |
| HC98 | 5 | Male | Chongqing | Community health control |
| HC99 | 4 | Male | Chongqing | Community health control |
| HC100 | 4 | Male | Chongqing | Community health control |
| HC101 | 5 | Male | Chongqing | Community health control |
| HC102 | 9 | Male | Chongqing | Community health control |
| HC103 | 3 | Female | Chongqing | Community health control |
| HC104 | 8 | Male | Chongqing | Community health control |
| HC105 | 4 | Female | Chongqing | Community health control |
| HC106 | 12 | Male | Chongqing | Community health control |
| HC107 | 4 | Male | Chongqing | Community health control |
| HC108 | 4 | Male | Chongqing | Community health control |
| HC109 | 3 | Male | Chongqing | Community health control |
| HC110 | 4 | Male | Chongqing | Community health control |
| HC111 | 4 | Female | Chongqing | Community health control |
| HC112 | 3 | Male | Chongqing | Community health control |
| HC113 | 3 | Male | Chongqing | Community health control |
| HC114 | 6 | Male | Chongqing | Community health control |
| HC115 | 3 | Female | Chongqing | Community health control |
| HC116 | 3 | Male | Chongqing | Community health control |
| HC117 | 3 | Male | Chongqing | Community health control |
| HC118 | 5 | Female | Chongqing | Community health control |
| HC119 | 2 | Male | Chongqing | Community health control |
| HC120 | 7 | Female | Chongqing | Community health control |
| HC121 | 2 | Male | Chongqing | Community health control |
| HC122 | 3 | Male | Chongqing | Community health control |
| HC123 | 12 | Male | Chongqing | Community health control |
| HC124 | 12 | Female | Chongqing | Community health control |
| HC125 | 4 | Male | Chongqing | Community health control |
| HC126 | 3 | Male | Chongqing | Community health control |
| HC127 | 5 | Male | Chongqing | Community health control |
| HC128 | 3 | Male | Chongqing | Community health control |
| HC129 | 4 | Male | Chongqing | Community health control |
| HC130 | 4 | Male | Chongqing | Community health control |
| HC131 | 5 | Female | Chongqing | Community health control |
| HC132 | 5 | Male | Chongqing | Community health control |
| HC133 | 2 | Male | Chongqing | Community health control |
| HC134 | 6 | Male | Chongqing | Community health control |
| HC135 | 3 | Female | Chongqing | Community health control |
| HC136 | 8 | Male | Chongqing | Community health control |
| HC137 | 4 | Female | Chongqing | Community health control |
| HC138 | 3 | Female | Chongqing | Community health control |
| HC139 | 7 | Male | Chongqing | Community health control |
| HC140 | 3 | Male | Chongqing | Community health control |
| HC141 | 5 | Female | Chongqing | Community health control |
| HC142 | 8 | Male | Chongqing | Community health control |
| HC143 | 8 | Male | Chongqing | Community health control |
| HC144 | 3 | Male | Chongqing | Community health control |
| HC145 | 4 | Female | Chongqing | Community health control |
| HC146 | 5 | Male | Chongqing | Community health control |
| HC147 | 4 | Male | Chongqing | Community health control |
| HC148 | 3 | Male | Chongqing | Community health control |
| HC149 | 4 | Female | Chongqing | Community health control |
| HC150 | 4 | Male | Chongqing | Community health control |
| HC151 | 9 | Male | Chongqing | Community health control |
| HC152 | 3 | Male | Chongqing | Community health control |
| HC153 | 4 | Male | Chongqing | Community health control |
| HC154 | 3 | Female | Chongqing | Community health control |
| HC155 | 4 | Male | Chongqing | Community health control |
| HC156 | 3 | Male | Chongqing | Community health control |
| HC157 | 3 | Female | Chongqing | Community health control |
| HC158 | 6 | Male | Chongqing | Community health control |
| HC159 | 5 | Female | Chongqing | Community health control |
| HC160 | 11 | Male | Chongqing | Community health control |
| HC161 | 3 | Male | Chongqing | Community health control |
