# Supplementary material for: Alteration of the gut microbiota profile in children with autism spectrum disorder in China
Source: Front Microbiol. 2024 Feb 13;14:1326870. doi: 10.3389/fmicb.2023.1326870 (PMC10899803; doi:10.3389/fmicb.2023.1326870)
Supplement: Supplementary Table S1 — Characteristics of study participants. [file Data_Sheet_4.PDF]

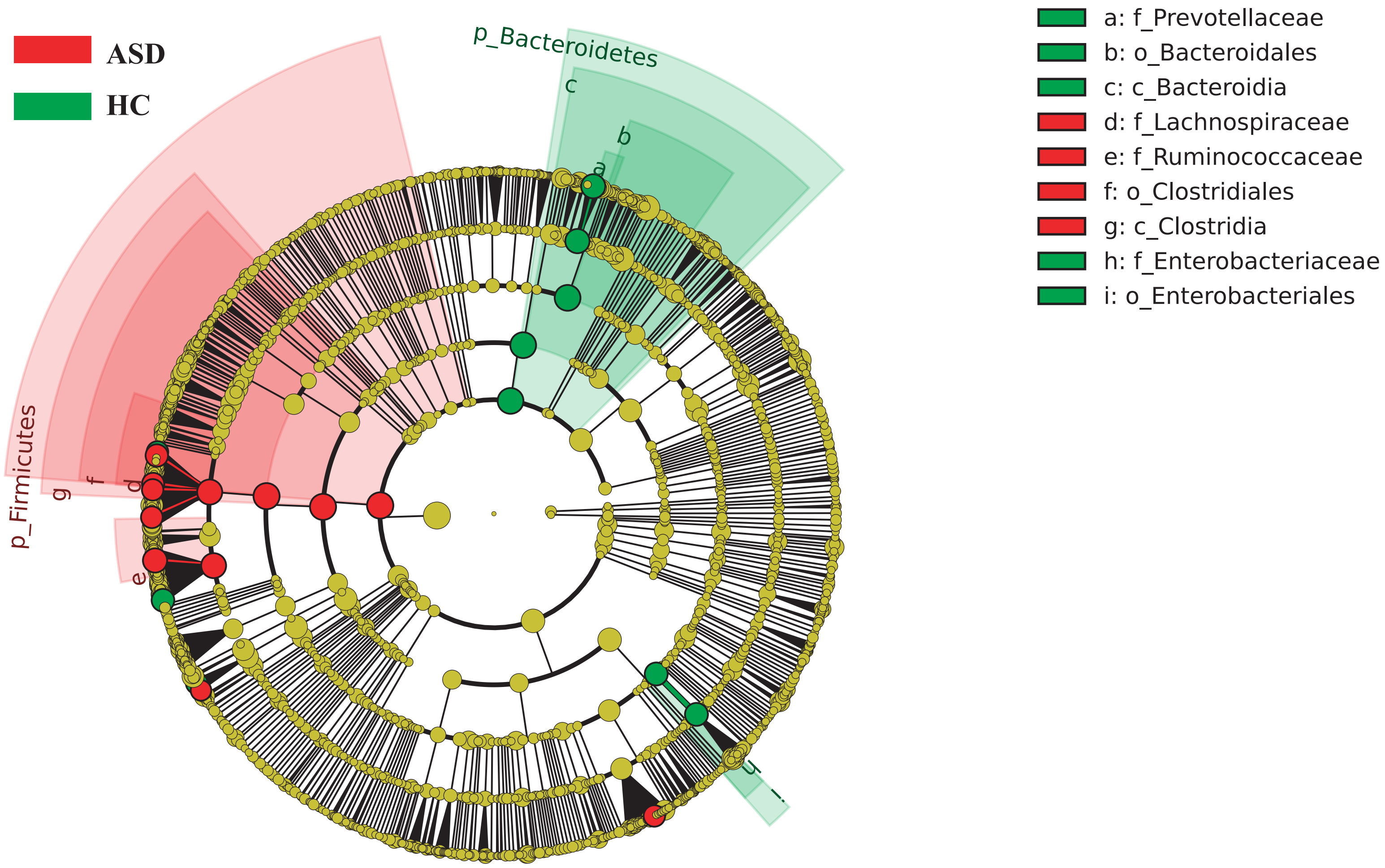
ASD
HC
p_Bacteroidetes
c
b
a
p_Firmicutes
g
f
d
e
a: f_Prevotellaceae
b: o_Bacteroidales
c: c_Bacteroidia
d: f_Lachnospiraceae
e: f_Ruminococcaceae
f: o_Clostridiales
g: c_Clostridia
h: f_Enterobacteriaceae
i: o_Enterobacteriales
